# Engineering auxin degradation into root-associated bacteria promotes plant growth

**DOI:** 10.1101/2025.10.25.684584

**Published:** 2025-10-26

**Authors:** Ting Jiang, Yihui Shen, Xi Li, Michal J. Kozlowski, Philip D. Jeffrey, John T. Groves, Joshua D. Rabinowitz, Jonathan M. Conway

**Affiliations:** 1 Department of Chemical and Biological Engineering, Princeton University, Princeton, NJ, USA.; 2 Department of Chemistry, Princeton University, Princeton, NJ, USA.; 3 Lewis-Sigler Institute for Integrative Genomics, Princeton University, Princeton, NJ, USA.; 4 Department of Molecular Biology, Princeton University, Princeton, NJ, USA.; 5 Ludwig Institute for Cancer Research, Princeton Branch, Princeton, NJ, USA.; 6 Omenn-Darling Bioengineering Institute, Princeton University, Princeton, NJ, USA.; 7 High Meadows Environmental Institute, Princeton University, Princeton, NJ, USA.; 8 Andlinger Center for Energy and the Environment, Princeton University, Princeton, NJ, USA.; 9 Present address: Department of Bioengineering, University of Pennsylvania, Philadelphia, PA, USA.; 10 These authors contributed equally: Ting Jiang, Yihui Shen

## Abstract

Overproduction of indole-3-acetic acid (IAA) by rhizosphere bacteria disrupts auxin homeostasis and induces root growth inhibition (RGI) in plants. Bacteria from the genus *Variovorax* mitigate this effect by degrading IAA, and in our prior work we identified the *iad* locus as being required for this activity. Here, we refine our understanding of the *iad* pathway using bacterial genetics, metabolomics, and isotope tracing to assign roles to individual Iad pathway enzymes and show that IadDE, though resembling a Rieske dioxygenase, functions instead as a monooxygenase that initiates catabolism via a novel intermediate. Guided by these insights, we installed chromosomal *iad* cassettes into root-associated commensals (*Polaromonas* MF047 and *Paraburkholderia* MF376), creating the first engineered bacteria that reprogram rhizosphere auxin homeostasis in microbially complex environments to benefit the plant. In natural soil, engineered *Paraburkholderia* enhanced plant biomass, and community profiling revealed no significant differences in microbiome composition between engineered and wild type treatments, supporting that auxin degradation conferred plant benefit without broader disruption of the rhizosphere community. Together, this work refines the pathway logic of microbial auxin degradation and demonstrates that commensals can be rationally engineered to deliver auxin-balancing functions in complex rhizosphere microbiomes. More broadly, it provides a framework for leveraging mechanistic insight to engineer plant-associated commensals that enhance plant growth, laying the foundation for deployment in agricultural settings.

## Introduction

Plant roots develop within a complex and densely populated microbial environment, where interactions with soil and rhizosphere microbes strongly influence root architecture and function^1,2^. A central regulator of root development is auxin, a group of signaling molecules that function as plant hormones but are also synthesized by bacteria, fungi, and animals^3^. Among them, indole-3-acetic acid (IAA) is the predominant and most indispensable auxin^4^. While plants maintain auxin homeostasis through biosynthesis, transport, conjugation, and degradation, many rhizosphere microbes also synthesize IAA. In microbial contexts, IAA contributes to bacterial physiology, stress adaptation, and microbe–microbe communication^3^. However, in plants, microbial IAA production can disrupt auxin homeostasis and lead to root growth inhibition (RGI)^5^. Genomic surveys indicate that over 80% of soil- and plant-associated bacterial genomes harbor complete or partial IAA biosynthetic pathways^6,7^, underscoring the widespread microbial influence on plant hormone dynamics.

Like a natural counterbalance, certain rhizosphere bacteria can mitigate RGI by degrading IAA. Two major types of auxin-degrading pathways have been identified in soil- or plant-associated bacteria: the *iad*-like and *iac*-like pathways^8,9^. The *iad*-like (IAA degradation) pathway, found in genera such as *Variovorax*, *Alcaligenes*, *Achromobacter* and *Bradyrhizobium*, converts IAA into anthranilic acid^5,9^. In contrast, the *iac*-like (indole-3-acetic acid catabolism) pathway, originally described in *Pseudomonas putida* 1290, degrades IAA into catechol^10,11^. Compared to the *iac*-like pathway, the *iad*-like pathway is more effective in reversing RGI induced by root-associated bacteria^9^. For example, *Variovorax* strains carrying the *iad* pathway fully restored root growth in *Arabidopsis thaliana* seedlings exposed to a 175-member RGI-inducing synthetic community, whereas *iac*-containing strains failed to do so^9^.

Despite its critical role, the molecular mechanisms underlying *iad*-mediated IAA degradation remain incomplete. The *iad* locus is known to be regulated by two MarR-family transcription factors, MarR73 and MarR50, with MarR73 serving as the primary repressor^9^. The first catalytic module, comprising IadDE (annotated as a Rieske non-heme dioxygenase) and the associated reductase IadC, is postulated to form a two-component dioxygenase system that facilitates electron transfer and enhances catalytic efficiency^9,12^.

Previous studies have demonstrated the feasibility of engineering IAA-degrading activity by introducing *iad* genes into heterologous hosts^5,9,12^. Plasmid-based expression of the *iad* locus in the root-associated isolate *Acidovorax* Root219 conferred IAA-degrading activity *in vitro* and promoted primary root elongation under exogenous IAA treatment^5^. However, this *Acidovorax* Root219 strain failed to fully reverse RGI caused by the auxin-producing *Arthrobacter* CL028, let alone more complex auxin-producing bacterial communities^5^. Additional knowledge of the contribution of *iad*-mediated auxin degradation to microbiome structure and mechanistic understanding of the genetics and biochemistry of the *iad* pathway would facilitate rational engineering of the *iad* locus into other strains. These past findings further suggest the best chassis strains would possess traits such as plant colonization capacity, competitiveness in the rhizosphere, and ecological stability as well as be genetically tractable for chromosomal genetic manipulation. Investigating new strains engineered with the *iad* pathway will lay the groundwork for future applications of the *iad* pathway and delivery of robust auxin homeostasis phenotypes in agricultural and ecological settings.

Here, we systematically dissect the *iad*-mediated IAA degradation pathway in *V. paradoxus* CL014 using a combination of genetic, metabolomics, and isotope tracing. We identify a nine-gene region (*iadCDEFGHIJK2*) within the *iad* locus that is responsible for IAA catabolism. *V. paradoxus* CL014 initiates IAA degradation through an unreported two-step oxidative mechanism. In the first step, the IadCDE complex functions as a monooxygenase that incorporates one oxygen atom from molecular oxygen into IAA. Although IadCDE is structurally homologous to canonical dioxygenase complexes, our metabolomic and isotopic analyses demonstrate that it functions as a monooxygenase, revealing a striking divergence between structure and catalytic mechanism. The second oxidative step is mediated by the dehydrogenase IadJ following hydrolysis, leading to oxygen incorporated from water. Notably, we identify the product of IadCDE to be an uncharacterized compound (C_10_H_9_NO_3_), rather than the commonly known intermediate, 2-oxindole-3-acetic acid (oxIAA, C_10_H_9_NO_3_), which is supportive of an epoxide-forming mechanism by IadCDE. To explore the broader applicability of this pathway, we genomically integrated the *iad* pathway genes, under control of the MarR73 regulator, into two additional root-associated bacteria, *Polaromonas* MF047 and *Paraburkholderia* MF376, and evaluated their IAA-degrading activity and ability to mitigate RGI in both simplified and complex bacterial community treatments, using *Arabidopsis thaliana* and *Medicago truncatula* as host plants. Engineered *Paraburkholderia* MF376 emerged as a promising chassis for agricultural applications, as it effectively reversed RGI caused by strains that *V. paradoxus* CL014 did not fully counteract, enhanced *Arabidopsis* growth in natural soil over long-term incubation, and exhibited robust colonization when introduced into new microbial communities. These findings advance our understanding of microbial auxin metabolism and its relevance to restoring rhizosphere auxin balance and enhancing plant growth.

## Results

### Identification of a nine-gene region within the *iad* locus responsible for IAA degradation in *Variovorax paradoxus* CL014

**Fig. 1 |:**
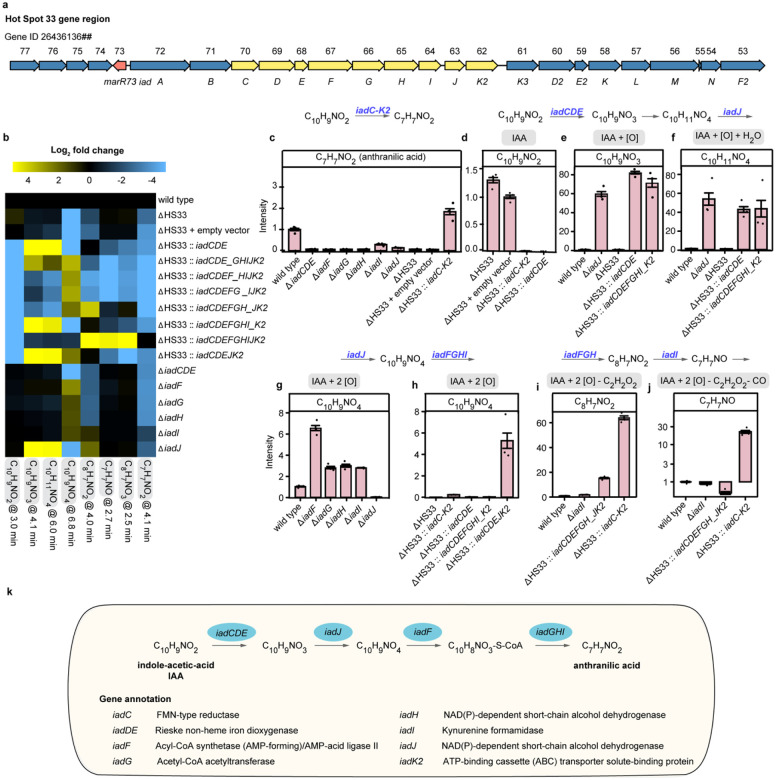
Metabolomic profiling of *iad* pathway mutants reveals substrates and products of genes involved in IAA degradation in *V. paradoxus* CL014. **a**, Genomic organization of Hot Spot 33 in *V. paradoxus* CL014. Gene annotations are shown above as the final two digits of the IMG gene ID (26436136##) and below with assigned gene names. The MarR-family transcriptional regulator *marR73* is shown in red; the core nine-gene IAA degradation locus is highlighted in yellow. **b**, Heatmap of Log_2_ fold changes in metabolite abundance in *iad* pathway mutants relative to the wild type. Full LC–MS profiles are provided in Extended Data Fig. 2. Data represent the mean of *n* = 3 biological replicates. **c–j**, Bar plots showing the relative abundance of anthranilic acid (**c**), IAA (**d**), and key metabolic intermediates (**e–j**) across wild-type and mutant strains. Data represent mean ± s.e.m. (standard error of the mean) of *n* = 3 biological replicates. **k**, Schematic representation of the *iad*-mediated IAA degradation pathway in *V. paradoxus* CL014.

Previous studies indicated that *V. paradoxus* CL014 degrades IAA through a pathway similar to that of *Bradyrhizobium japonicum*, producing intermediates such as dioxindole, isatin, isatinic acid, and anthranilic acid—an intermediate in tryptophan biosynthesis^9,13,14^. However, the specific genes responsible for each step remained unidentified. The *iad* locus (Hot Spot 33, HS33), consisting of 25 genes (Fig. 1a), was identified as responsible for IAA degradation via genomic deletion^5^. To pinpoint key genes, we cloned different segments of the *iad* locus into the broad-host vector pBBR1 and introduced them into an *iad* locus deletion mutant (ΔHS33) (Extended Data Fig. 1a). Metabolomic analysis revealed that mutants carrying the *iadC-K2* region restored the level of intermediates and the final product, anthranilic acid, indicating that this nine-gene region encodes the complete IAA degradation pathway (Fig.1a, Extended Data Fig. 1b).

To identify specific substrates and products of *iad* genes, we performed liquid chromatography in tandem with high-resolution mass spectrometry (LC-MS), focusing on metabolites previously associated with IAA degradation in *V. paradoxus* CL014^9^. We reasoned that knocking out a gene downstream of a metabolite or overexpressing a gene upstream would lead to metabolite accumulation or depletion, respectively. Specifically, we created knockout mutants in the wild-type background and overexpression mutants in the ΔHS33 background (Fig. 1b, Extended Data Fig. 2). Based on gene annotations and structural data, *iadD* and *iadE* encode the large and small subunits of a Rieske non-heme dioxygenase, while *iadC* encodes a reductase, forming a two-component dioxygenase system (Fig. 1k)^9,12^. Accordingly, *iadCDE* was treated as a single functional unit for mutant construction.

Our analysis revealed that deletion of *iadCDE*, *iadF*, *iadG*, *iadH*, *iadI*, or *iadJ* significantly reduced anthranilic acid (C_7_H_7_NO_2_) production (Fig. 1c), confirming their essential roles in IAA degradation. Complementation of the ΔHS33 mutant with the full *iadC–K2* gene region fully restored anthranilic acid levels (Fig. 1c). Notably, the presence of *iadCDE* in these segments was able to rapidly degrade IAA. This confirms that IadCDE directly uses IAA as its substrate and catalyzes the initial step of IAA degradation (Fig. 1d, Extended Data Fig. 1c). We next examined metabolite accumulation in the ΔHS33::*iadCDE* strain and identified two compounds, C_10_H_9_NO_3_ and likely its hydration product C_10_H_11_NO_4_, suggesting C_10_H_9_NO_3_ to be the product of IadCDE (Fig. 1e, f). Interestingly, *iadJ* deficiency also led to the accumulation of C_10_H_9_NO_3_ and C_10_H_11_NO_4_, indicating that IadJ likely consumes one of these intermediates (Fig. 1e). Similarly, we found that C_10_H_9_NO_4_ is depleted with *iadJ* deletion while it accumulates in ΔHS33::*iadCDEJK2*, suggesting C_10_H_9_NO_4_ to be the product of IadJ (Fig. 1g, h). Deletion of *iadF*, *iadG*, *iadH* and *iadI* all lead to C_10_H_9_NO_4_ accumulation, among which Δ*iadF* shows the strongest accumulation suggesting functional proximity of IadF to IadCDEJ (Fig. 1g). Other projected intermediates were not detected, thus IadFGHI likely carry out a chain of reactions, with most intermediates quickly channeled through the enzymes. Therefore, we resorted to annotated enzyme function to infer reactions carried out by these genes. Annotated as an acyl-CoA synthase (AMP-forming), IadF likely activates the carboxylic group in the acetyl side chain, enabling its subsequent removal by IadG, an acetyl-CoA acetyltransferase (ketothiolase, EC 2.3.1.16) (Fig. 1k). This reaction yields CoA-bound intermediates that are membrane-impermeable, ensuring their retention within bacterial cells and enabling efficient recognition and processing^14^. Meanwhile, IadH, annotated as an alcohol dehydrogenase, likely facilitates further processing (Fig. 1k). Together, IadF, IadG, and IadH mediate the reductive removal of the acetyl side chain (-C_2_H_2_O_2_), producing C_8_H_7_NO_2_ (Fig. 1i). Indeed, C_8_H_7_NO_2_ accumulates in ΔHS33::*iadCDEFGH*_*JK2* expression strain as well as the *iadI* deletion strain (Fig. 1i), suggesting that IadI acts downstream of C_8_H_7_NO_2_. We also detected C_7_H_7_NO and C_8_H_7_NO_3_, which strongly accumulate only in strains overexpressing the whole *iadC-K2* gene locus, suggesting that they are likely intermediates between the IadI product and the final product anthranilic acid (C_7_H_7_NO_2_) (Fig. 1b, j). Although *iadI* is annotated as a kynurenine formamidase, our MS/MS analysis confirmed that C_8_H_7_NO_3_ is not N-formylanthranilic acid—the expected formylated derivative of anthranilic acid—indicating that IadI may instead function as a broad-specificity amidase. Lastly, IadK2 was previously identified as a highly IAA-specific ATP-binding cassette (ABC) transporter solute-binding protein involved in IAA uptake^12^. However, it is not essential, as ΔHS33::*iadCDE* was still capable of taking up and utilizing IAA, suggesting alternative uptake mechanisms^9^.

In summary, we identified a nine-gene region (*iadC-K2*) within the *iad* locus responsible for IAA degradation in *V. paradoxus* CL014 (Fig. 1a, Extended Data Fig. 1a, b). By integrating metabolite abundance changes with gene functional annotations, we assigned putative reaction steps to each gene and reconstructed the degradation pathway (Fig. 1k). This pathway links specific functional genes to corresponding intermediates, providing a framework for understanding IAA metabolism in this microorganism.

### Isotope tracing reveals new intermediates and an unreported two-step oxidative mechanism revising the *iad*-mediated IAA degradation pathway

Previously, IAA degradation via the *iad* pathway was proposed to proceed through monooxygenation to generate 2-oxindole-3-acetic acid (oxIAA, C_10_H_9_NO_3_), followed by conversion to dioxindole^9,12^. Our metabolomic analysis, however, revealed two distinct C_10_H_9_NO_3_ peaks in wild-type extracts: a major peak at 4 min and a minor peak at 7 min (Extended Data Fig. 3a). Using an oxIAA standard, we confirmed that the minor peak corresponds to oxIAA (Extended Data Fig. 3a); however, this compound is not depleted in the ΔHS33 mutant, making it unlikely to be the product of IadCDE (Extended Data Fig. 3b–c). By contrast, the major C_10_H_9_NO_3_ peak is depleted in the ΔHS33 mutant and accumulates in *iadCDE* overexpressing strains, indicating that it represents the product of IadCDE (Extended Data Fig. 3b–d). These findings indicate that the observed C_10_H_9_NO_3_ is not oxIAA but rather a previously uncharacterized intermediate, suggesting that IAA degradation in *V. paradoxus* CL014 proceeds via a mechanism distinct from the canonical oxIAA pathway.

**Fig. 2 |:**
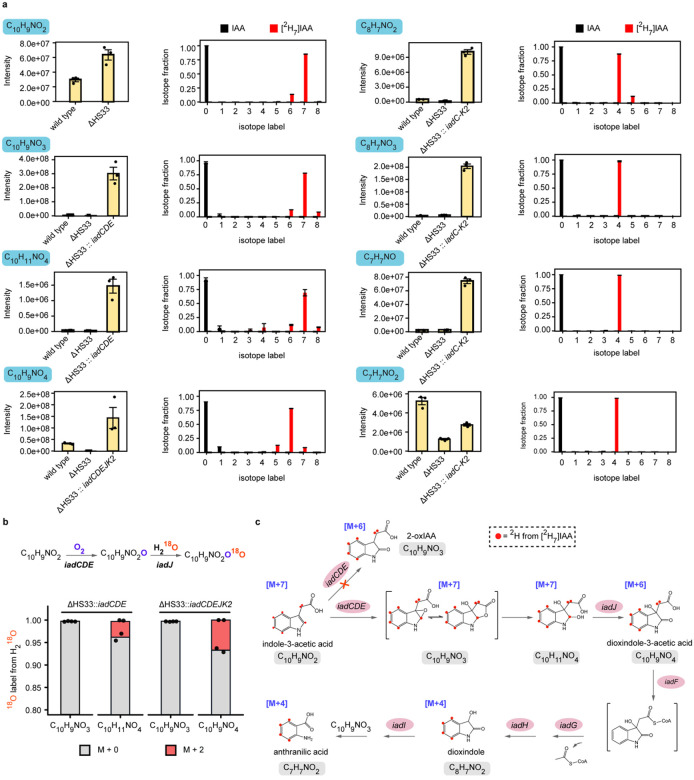
Isotope tracing with [^2^H_7_]IAA and H218O reveals a revised IAA degradation pathway in *V. paradoxus* CL014. **a**, LC-MS analysis of [^2^H_7_]IAA catabolism identifies key pathway intermediates and their deuterium labeling profiles. Left, LC–MS peak intensities of major metabolites. Right, deuterium isotope distributions following incubation with unlabeled IAA or [^2^H_7_]IAA. Data represent mean ± s.e.m. (standard error of the mean) of *n* = 3 biological replicates. **b**, H_2_^1^ O (20% v/v) tracing confirms the source of oxygen atoms incorporated during the two-step oxidation reactions. Data are shown for two biological replicates. **c**, A revised mechanistic model of IAA degradation consistent with isotope tracing.

To elucidate the mechanism of IAA degradation, we performed isotope tracing and LC-MS/MS analysis of both labeled and unlabeled pathway intermediates (Fig. 2, Extended Data Fig. 3 and 4). Specifically, we employed uniformly deuterium-labeled IAA ([^2^H_7_]IAA) to monitor hydrogen rearrangements during catabolism, providing greater mechanistic resolution than the previously used [^13^C_6_]IAA^9^. Tracing with [^2^H_7_]IAA revealed distinct mass shifts in pathway intermediates relative to unlabeled controls, indicating the number of retained deuterium atoms. The first intermediate, C_10_H_9_NO_3_, retained all seven deuterium atoms, suggesting no isotope loss during the initial oxidation step (Fig. 2a, Extended Data Fig. 4a, c). This observation is inconsistent with mechanisms involving oxIAA or its enol form, 2-hydroxy-IAA^9,12^, or a radical intermediate from hydrogen atom abstraction^15^. Indeed, the minor C_10_H_9_NO_3_ peak corresponding to oxIAA retained only six deuterium atoms (Extended Data Fig. 3d). The subsequent intermediate, C_10_H_9_NO_4_, showed the loss of one deuterium, whereas all downstream intermediates consistently retained four deuterium atoms on the aromatic ring (Fig. 2a, Extended Data Fig. 4a, c).

To identify the source of oxygen in the formation of C_10_H_9_NO_3_, C_10_H_11_NO_4_, and C_10_H_9_NO_4_, respectively, we performed ^1^ O-labeled water (H_2_^1^ O) tracing in bacterial culture (Fig. 2b, Extended Data Fig. 4b). The IadCDE monooxygenase complex uses molecular oxygen (O_2_) and therefore will not generate labeled product (C_10_H_9_NO_3_) from H_2_^1^ O. On the contrary, the IadJ dehydrogenase mechanism leads to oxidized product that can be labeled by H_2_^1^ O. The minor C_10_H_9_NO_3_ peak corresponding to oxIAA is significantly labeled by H_2_^1^ O, further confirming oxIAA is not formed by IadCDE (Extended Data Fig. 3e). In contrast, we detected no labeling in the major C_10_H_9_NO_3_ peak in *iadCDE* overexpressing strain, consistent with it being the product of IadCDE (Fig. 2b). Meanwhile, both C_10_H_11_NO_4_ and C_10_H_9_NO_4_ incorporated ^1^ O in the same strains, supporting that C_10_H_11_NO_4_ is formed via hydration of C_10_H_9_NO_3_ in the cells, and is further processed by IadJ to C_10_H_9_NO_4_ through a dehydrogenase mechanism (Fig. 2b). Further MS/MS analysis of C_10_H_9_NO_4_ revealed that the incorporated ^1^ O resides either on the carboxylate or the ketone moiety (Extended Data Fig. 4b–d). Interestingly, C_10_H_9_NO_4_ remained almost unlabeled in the wild type, which may be due to the rapid hydrolysis of C_10_H_9_NO_3_ within the IadCDE catalytic center, where H_2_O is derived from catalytic reduction of molecular oxygen possibly by IadC.

Collectively, our data support a revised IAA degradation pathway in *V. paradoxus* CL014 (Fig. 2c). Specifically, the initial oxidation of IAA proceeds through two sequential steps (C_10_H_9_NO_2_ → C_10_H_9_NO_3_ → C_10_H_9_NO_4_) (Fig. 2c). IadCDE functions as a monooxygenase complex that carries out the first oxidation, incorporating oxygen derived from molecular oxygen, whereas IadJ acts as a dehydrogenase that completes the second oxidation, incorporating the oxygen atom from H_2_O. Our data reveal that the intermediate C_10_H_9_NO_3_ is distinct from commonly believed pathway intermediate, oxIAA, although they share the same molecular formula. Following the observed deuterium labeling and mechanisms of known heme-dependent tryptophan or indole dioxygenases^16–19^, we propose that oxidation by IadCDE proceeds via a 2,3-epoxide intermediate. This is followed by spontaneous ring opening via hydrolysis and IadJ catalyzed dehydrogenation to produce dioxindole-3-acetic acid (C_10_H_9_NO_4_). This revised pathway provides a more accurate framework for understanding IAA degradation in *V. paradoxus* CL014. By homology, it suggests that the *iad*-like pathway represents a conserved and unique auxin-catabolic strategy among certain plant-associated commensals^9,20^. This work also lays the foundation for further exploration of *iad*-like pathways in these plant-associated bacteria in genera such as *Bradyrhizobium*, *Alcaligenes*, and *Achromobacter*^9^.

### The structurally and functionally divergent oxygenase complex IadCDE reverses bacteria-induced root growth inhibition

**Fig. 3 |:**
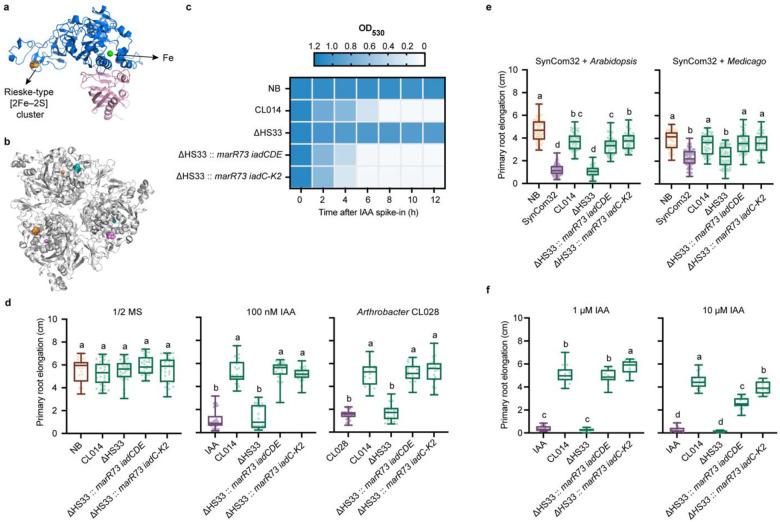
Structural and genetic evidence show that IadCDE enables auxin degradation and alleviates root growth inhibition. **a**, Crystal structure of the IadDE complex from *V. paradoxus* CL014 resolved at 1.28 Å (PDB code: 9O71). **b**, A heterohexameric IadDE complex is formed by trimerization of IadDE heterodimers. The Rieske-type [2Fe–2S] cluster and mononuclear iron within each IadD subunit are shown in matching colors. Trimer assembly reduces the spatial distance between the electron donor (Rieske cluster) and acceptor (iron center), potentially enhancing catalytic electron transfer efficiency. **c**, *in vitro* IAA degradation by engineered strains. IAA concentrations were measured from culture supernatants of strains grown in M9 minimal medium supplemented with glucose and 0.1 mg/mL IAA. The Salkowski reagent reacts with IAA to generate a pink-to-red chromophore with an absorption maximum at 530 nm (OD_530_). Data represent the mean of two biological replicates per sample. **d–f**, Primary root length of *Arabidopsis* seedlings exposed to 100 nM IAA or the auxin-producing strain *Arthrobacter* CL028 (**d**), A 32-member synthetic community (SynCom32) in *Arabidopsis* and *Medicago* seedlings (**e**), and elevated IAA concentrations (1 μM and 10 μM) in *Arabidopsis* (**f**). Letters above boxplots indicate statistically significant differences as determined by one-way ANOVA with Tukey’s post hoc test (*P* < 0.05). Groups not sharing the same letter differ significantly. “NB” indicates the no-bacteria control. Sample sizes (left to right): **d**, *n* = 23, 29, 31, 31, 27, 34, 26, 34, 26, 25, 31, 16, 21, 21, 25; **e**, *n* = 64, 81, 56, 52, 53, 43, 52, 78, 52, 71, 47, 48; **f**, *n* = 19, 15, 18, 14, 9, 19, 17, 19, 19, 14. Box plots represent the median (center line), interquartile range (box), and 1.5× interquartile range (whiskers).

Oxygenases play a crucial role in aerobic bacteria by incorporating oxygen into chemically stable aromatic compounds, enabling their degradation into metabolically accessible substrates^14,21^. To date, the most extensively studied indole oxygenases fall into two major families: the tryptophan dioxygenase (TDO) superfamily, which catalyzes dioxygenation or monooxygenation via an Fe/heme-dependent mechanism^18,19,22^; and the flavin-dependent monooxygenases (FMOs), which use a flavin cofactor to mediate oxygen transfer^23–25^. Rieske-type dioxygenases may function divergently as dioxygenase, monooxygenase, or hydroxylase^15–17^. Our metabolomics and isotope tracing reveal a monooxygenation-driven mechanism, identifying IadCDE in *V. paradoxus* CL014 as a two-component indole monooxygenase, essential for initiating IAA degradation, rather than a dioxygenase as previously annotated^9,12^. The proposed reaction mechanism closely resembles that of MarE, a heme-dependent aromatic monooxygenase involved in the maremycin biosynthetic pathway of *Streptomyces sp*. B9173^17–19,22^. While MarE belongs to the TDO superfamily and relies on a heme cofactor^17,18^, IadDE is structurally similar to Rieske non-heme dioxygenases (Fig. 3a, b)^12,15^.

To investigate the molecular basis of IadCDE function, we determined the crystal structure of the IadDE complex (PDB code: 9O71) from *V. paradoxus* CL014 at 1.28 Å resolution, providing a high-resolution view of the complete holoenzyme. While a homologous structure was previously resolved by cryo-EM at 1.8 Å resolution (with 98.4% and 100% protein sequence identity for IadD and IadE, respectively)^12^ the improved resolution of our X-ray structure allows for more precise visualization of overall structural features. Structurally, IadDE adopts the canonical fold of Rieske-type non-heme dioxygenases^26^. IadD and IadE assemble into a heterodimeric α_3_β_3_ quaternary complex with threefold symmetry and a characteristic mushroom-shaped morphology (Fig. 3b)^12^. In the IadCDE complex, IadD contains both a Rieske-type [2Fe–2S] cluster and a mononuclear iron center and serves as the catalytic subunit, while IadE provides structural support. Trimerization of IadDE heterodimers brings the Rieske iron-sulfur cluster of IadD close to the iron center of an adjacent IadD, potentially facilitating electron transfer during catalysis (Fig. 3b). IadC is annotated as a reductase and is proposed to mediate electron transfer from NADH to IadD to activate molecular oxygen^12^.

Phylogenetic analysis revealed that IadD forms a distinct subclade within the Rieske dioxygenase and indole oxygenase family, showing close evolutionary relationships to the phthalate family of Rieske non-heme dioxygenases^27^, as well as to members of the TDO and FMO superfamilies. This positioning may reflect shared functional features among distinct oxygenase lineages (Extended Data Fig. 5).

To determine whether *iadCDE* alone is sufficient to reverse RGI in the context of complex microbial communities, we genomically integrated the native repressor *marR73*^9^ alongside either the minimal^9^ (*iadCDE*) or full (*iadC-K2*) degradation pathway into the *Variovorax* ΔHS33 strain. This enabled direct functional comparison of the oxygenase module with the complete pathway under IAA-rich conditions. In M9 minimal medium supplemented with glucose and 0.1 mg/mL IAA, both engineered strains showed significantly enhanced IAA degradation relative to the wild type, independent of bacterial growth (Fig. 3c, Extended Data Fig. 6a). In a gnotobiotic system, engineered strains fully rescued RGI in *Arabidopsis* seedlings challenged with 100 nM exogenous IAA, the auxin-producing strain *Arthrobacter* CL028, or a 32-member synthetic community (SynCom32, Supplementary Table 1), with comparable effects observed in *Medicago* (Fig. 3d, e). These results demonstrate that *iadCDE* alone is sufficient to reverse RGI caused by both exogenous IAA and auxin-producing microbes. However, under elevated IAA concentrations (1 μM or 10 μM), the strain harboring the full degradation pathway outperformed the minimal mutant, suggesting that downstream enzymatic steps confer an advantage in detoxifying excess IAA (Fig. 3f).

### Engineering of *iad* genes into two distinct root-associated bacteria confers IAA-degrading activity and promotes auxin homeostasis

**Fig. 4 |:**
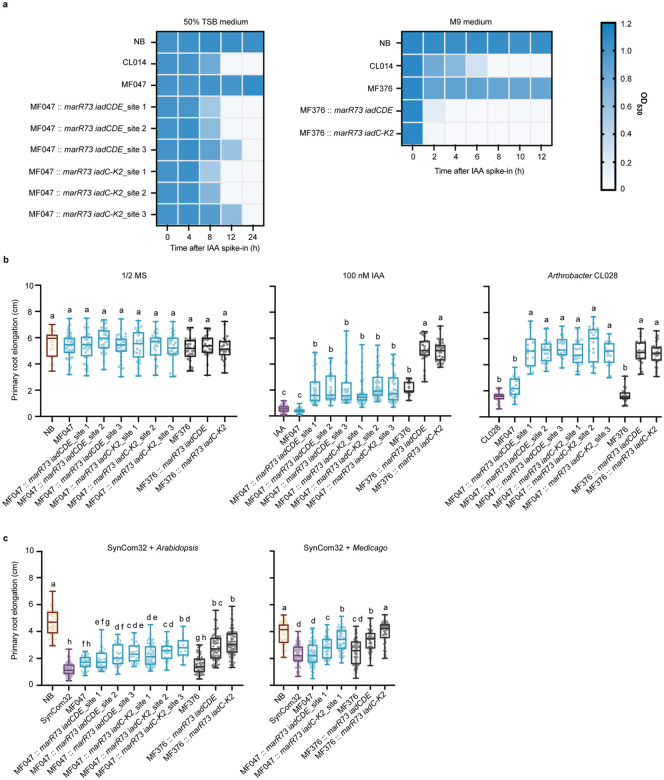
Engineering root-associated bacteria with *iad* genes enables IAA degradation and alleviates root growth inhibition (RGI). **a**, Engineered *Polaromonas* MF047 strains cultured in 50% TSB medium and *Paraburkholderia* MF376 strains cultured in M9 medium efficiently degraded IAA. IAA levels were quantified from culture supernatants using the Salkowski reagent, which forms a pink-to-red chromophore upon reaction with IAA and is measured at OD_530_. Data represent the mean of two biological replicates per sample. **b–c**, Engineered chromosomal knock-in strains reversed RGI, as indicated by enhanced primary root elongation in *Arabidopsis* seedlings treated with 100 nM IAA or the auxin-producing strain *Arthrobacter* CL028 (**b**), and in *Arabidopsis* and *Medicago* seedlings exposed to a 32-member synthetic bacterial community (SynCom32) (**c**). Letters above boxplots indicate statistically significant differences as determined by one-way ANOVA with Tukey’s post hoc test (*P* < 0.05). Groups not sharing the same letter differ significantly. “NB” denotes the no-bacteria control. Sample sizes (left to right): **b**, *n* = 23, 42, 37, 31, 34, 26, 30, 27, 31, 26, 25, 51, 42, 44, 33, 39, 52, 49, 42, 26, 28, 35, 31, 21, 15, 21, 21, 23, 21, 17, 22, 22, 29; **c**, *n* = 64, 81, 45, 41, 33, 28, 61, 36, 17, 56, 75, 89, 52, 78, 76, 58, 67, 74, 57, 68. Box plots show the median (horizontal line), interquartile range (boxes), and whiskers extending to 1.5× the interquartile range.

To evaluate whether root-associated bacteria can acquire IAA-degrading activity through genetic engineering, we introduced *iad* genes into two root-associated strains: *Polaromonas* MF047 and *Paraburkholderia* MF376^28^. Both strains, like *Variovorax*, are Betaproteobacteria and do not impair host growth when co-inoculated with *Arabidopsis* seedlings (Fig. 4b). Neither strain produced IAA when cultured in M9 minimal medium supplemented with tryptophan and glucose, nor were they able to reverse RGI triggered by exogenous IAA or by the auxin-producing strain *Arthrobacter* CL028 (Fig. 4b, Extended Data Fig. 6d). Additionally, as an amino acid auxotroph, *Polaromonas* MF047 exhibited no growth in M9 minimal medium unless supplemented with amino acids (Extended Data Fig. 6e).

To confer IAA-degrading capacity, we genomically integrated *marR73* together with either the minimal (*iadCDE*) or complete (*iadC-K2*) pathway into three intergenic sites in *Polaromonas* MF047 and one site in *Paraburkholderia* MF376 (Extended Data Fig. 6b). To minimize potential disruption of native gene expression, insertion sites were located within non-coding intergenic regions ranging from 400 to 900 bp in length. All engineered strains degraded IAA within 24 hours, and *Paraburkholderia* MF376 knock-ins achieved complete IAA removal within four hours (Fig. 4a). This rapid degradation alleviated RGI in *Arabidopsis* seedlings exposed to 100 nM exogenous IAA (Fig. 4b). Growth curves in 50% TSB medium revealed no significant differences between wild-type and engineered strains, indicating that chromosomal integration did not impair bacterial fitness (Extended Data Fig. 6a).

To assess their function in plant-microbe interactions, we co-inoculated engineered strains with *Arthrobacter* CL028 or SynCom32 on 7-day-old *Arabidopsis* seedlings in a gnotobiotic system. After 7 days, all engineered strains fully rescued RGI triggered by *Arthrobacter* CL028, restoring primary root length to control levels (Fig. 4b). In parallel, they significantly alleviated RGI induced by SynCom32, with *Paraburkholderia* MF376 consistently showing greater efficacy than *Polaromonas* MF047 (Fig. 4c). To test cross-species efficacy, we repeated the SynCom32 experiment using *Medicago* seedlings. In this context, strains carrying the full *iad* pathway promoted stronger root growth than those expressing only the minimal *iadCDE* module, highlighting the importance of a complete IAA degradation pathway for effective function in complex microbial environments (Fig. 4c). These results demonstrate for the first time that stable genomic integration and expression of *iad* genes in root-associated bacteria is able to restore root growth under microbial auxin stress. The engineered strains are effective across microbial contexts and plant hosts, underscoring their potential as tools for microbiome-based modulation of plant development.

### Engineered *Paraburkholderia* MF376 reverses RGI that *V. paradoxus* CL014 cannot fully rescue

**Fig. 5 |:**
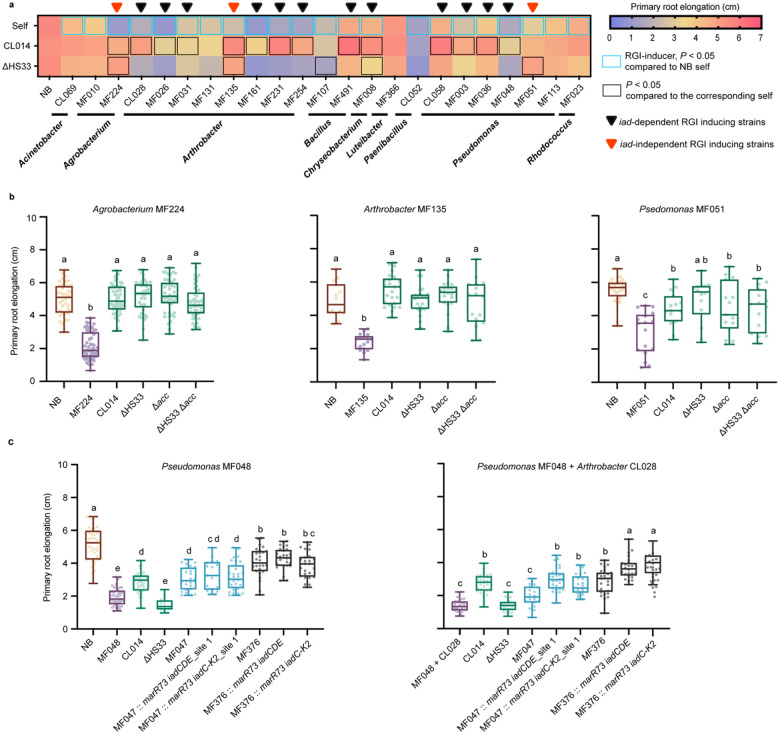
Root growth inhibition by certain strains is mitigated independently of the *iad* locus, while engineered *Paraburkholderia* MF376 enhances mitigation of *iad*-dependent inhibition. **a**, Heatmap showing average primary root lengths of *Arabidopsis* seedlings inoculated with 23 previously identified RGI-inducing strains^5^, either alone (self) or co-inoculated with *V. paradoxus* CL014 wild type or the *iad*-deficient mutant ΔHS33, to assess *iad*-dependent reversion. Blue squares indicate significant differences compared to the no-bacteria (NB) control demonstrating RGI phenotypes; black squares indicate significant differences compared to the strain inoculated alone (self). Corresponding box plots are shown in Extended Data Fig. 7. **b**, Primary root length of *Arabidopsis* seedlings co-inoculated with *iad*-independent RGI-inducing strains and treated with *V. paradoxus* CL014 wild type, ΔHS33, Δ*acc* (ACC deaminase gene deleted), or ΔHS33 Δ*acc* (both *iad* and ACC deaminase genes deleted). **c**, Primary root lengths of *Arabidopsis* seedlings inoculated with *Pseudomonas* MF048 alone or with *Arthrobacter* CL028, and co-inoculated with *V. paradoxus* CL014, or *iad*-engineered strains of *Polaromonas* MF047 or *Paraburkholderia* MF376. Letters above boxplots indicate statistically significant differences as determined by one-way ANOVA with Tukey’s post hoc test (*P* < 0.05). Groups not sharing the same letter differ significantly. “NB” denotes the no-bacteria control. Sample sizes (left to right): **b**, *n* = 40, 56, 48, 41, 48, 49, 14, 17, 25, 25, 21, 16, 28, 17, 15, 14, 15, 15; **c**, *n* = 42, 41, 41, 17, 25, 17, 29, 27, 29, 25, 26, 28, 22, 19, 30, 21, 30, 24, 25. Box plots show the median (center line), interquartile range (boxes), and whiskers extending to 1.5× the interquartile range.

To assess the contribution of the *iad* pathway to RGI mitigation, we selected 23 previously identified RGI-inducing strains from nine bacterial genera (Supplementary Table 1)^5^. Each strain was co-inoculated with either wild-type *V. paradoxus* CL014 or the *iad* deletion mutant ΔHS33 on 7-day-old *Arabidopsis* seedlings. After 7 days, root length measurements revealed that 22 strains induced RGI (Fig. 5a, b; Extended Data Fig. 7). Among these, RGI caused by 16 strains was reversed by wild-type *V. paradoxus* CL014, and in 12 of these cases (12/16), the wild type restored root growth more effectively than the *iad*-deficient mutant ΔHS33, indicating an *iad*-dependent mitigation mechanism (Extended Data Fig. 7). In contrast, for *Agrobacterium* MF224, *Arthrobacter* MF135, and *Pseudomonas* MF051, both wild-type and ΔHS33 strains restored root growth to similar levels, suggesting an *iad*-independent mechanism (Fig. 5b).

Genome analysis of *V. paradoxu*s CL014 revealed a gene encoding 1-aminocyclopropane-1-carboxylic acid (ACC) deaminase, an enzyme that degrades ACC, the ethylene precursor^29^. Ethylene, a plant hormone, can inhibit primary root elongation at high levels^30^. Prior studies show that RGI induced by *Arthrobacter* CL028 and a synthetic microbial community requires both auxin and ethylene signaling in the host^5^. While plants produce ethylene endogenously, certain soil and rhizosphere microbes also contribute to ethylene levels^29^. Notably, some *Agrobacterium*, *Arthrobacter*, and *Pseudomonas* strains have been reported to produce ethylene, which can suppress root elongation and alter root architecture^31–33^. Based on these observations, we hypothesized that the three *iad*-independent strains may produce both auxin and ethylene, with IAA degradation mediated by the *iad* pathway and ethylene detoxification potentially supported by ACC deaminase activity in *V. paradoxus* CL014. However, deletion of the ACC deaminase gene in both wild-type and ΔHS33 backgrounds did not impair their ability to reverse RGI, ruling out ethylene degradation through this ACC deaminase as the primary mechanism (Fig. 5b). These findings suggest that while the *iad* pathway is a central mechanism for suppressing RGI caused by many root-associated strains, *V. paradoxus* CL014 also employs *iad*-independent strategies to mitigate RGI from certain microbes.

Despite its broad effectiveness, *V. paradoxus* CL014 was unable to fully rescue RGI induced by *Pseudomonas* MF048. To test whether *iad*-engineered strains could provide enhanced reversion, we evaluated *Polaromonas* MF047 and *Paraburkholderia* MF376 engineered with the *iad* pathway. These strains were co-inoculated with *Pseudomonas* MF048 alone or in combination with *Arthrobacter* CL028 on *Arabidopsis* seedlings. Root length analysis showed that *Paraburkholderia* MF376 knock-in strains significantly outperformed both *V. paradoxus* CL014 and engineered *Polaromonas* MF047 under both conditions (Fig. 5c). Collectively, these results demonstrate that engineering *Paraburkholderia* MF376 with the *iad* pathway enhances its ability to mitigate complex RGI interactions, highlighting its potential as a robust and versatile chassis for promoting plant growth in diverse microbial environments.

### Impact of *V. paradoxus* CL014 and engineered strains on the root microbiome and plant growth

**Fig. 6 |:**
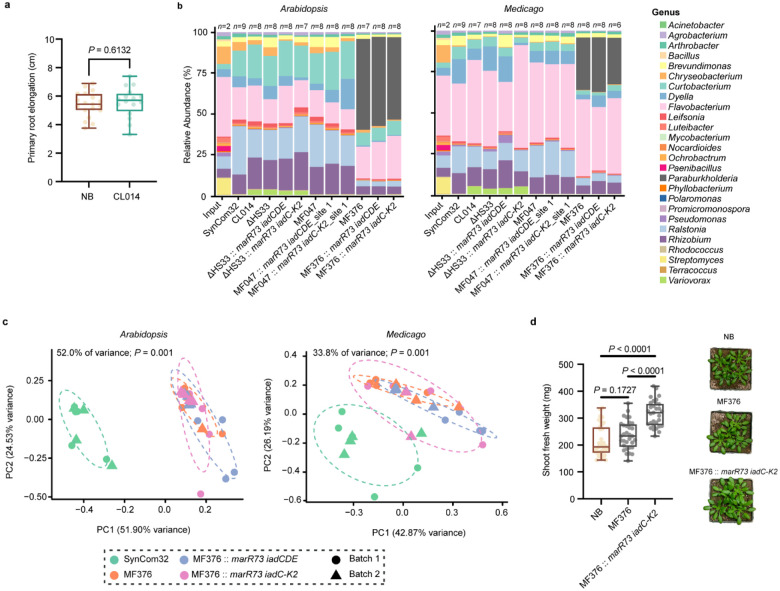
Engineered strains do not alter root microbiome composition but promote Arabidopsis growth. **a**, Primary root length of *Arabidopsis* seedlings treated with *V. paradoxus* CL014 at OD_600_ = 2. Statistical significance was assessed using Welch’s two-tailed *t*-test (*n* = 19, 16). **b**, Relative abundance of bacterial genera in the root microbiomes of *Arabidopsis* and *Medicago* seedlings treated with SynCom32 in combination with wild-type or *iad*-engineered strains of *V. paradoxus* CL014, *Polaromonas* MF047, and *Paraburkholderia* MF376. Sample sizes are indicated above each bar. **c**, Unconstrained principal coordinate analysis (PCoA) of Bray–Curtis dissimilarity showing root microbiome profiles in *Arabidopsis* and *Medicago* seedlings treated with SynCom32 alone or with engineered *Paraburkholderia* MF376 strains. Ellipses represent 68% confidence intervals. Statistical significance was determined by PERMANOVA (Adonis2). **d**, Shoot fresh weight of 33-day-old *Arabidopsis* plants grown in untreated natural soil inoculated individually with *Paraburkholderia* MF376 wild type and *marR73 iadC-K2* knock-in strains. Statistical significance was determined using one-way ANOVA with Tukey’s post hoc test (*n* = 36, 32, 31). Data shown in panels **a–d** are derived from two independent experiments.

*V. paradoxus* CL014 can degrade IAA without harming plant growth at normal treatment levels (OD_600_ = 0.05). To test whether this effect holds under high bacterial load, we applied *V. paradoxus* CL014 at OD_600_ = 2 to *Arabidopsis* seedlings in a gnotobiotic system. No significant changes in primary root length were observed, indicating that even at high densities, *V. paradoxus* CL014 does not adversely affect plant growth or disturb auxin balance (Fig. 6a). These findings suggest that its IAA-degrading function may primarily influence microbe–microbe interactions rather than directly altering plant development.

To assess the impact of *V. paradoxus* CL014, *Polaromonas* MF047, *Paraburkholderia* MF376, and their *iad*-gene-containing engineered strains on the root microbiome, we treated *Arabidopsis* and *Medicago* seedlings with SynCom32 (Supplementary Table 1) in the presence or absence of each strain. After 9 days, root-associated communities were profiled by 16S rRNA amplicon sequencing. Despite receiving the same SynCom32 inoculum, *Arabidopsis* and *Medicago* developed distinct root microbiomes, highlighting the influence of host genotype (Fig. 6b). Among the three introduced genera, *Paraburkholderia* exhibited the highest relative abundance in roots (50.84%), followed by *Variovorax* (3.40%), whereas *Polaromonas* (0.01%) showed minimal colonization (Fig. 6b, and Supplementary Table 6). Due to its robust root colonization, *Paraburkholderia* MF376—both wild-type and engineered—substantially altered root microbiome composition, explaining 52% and 33.8% of the community variation in *Arabidopsis* and *Medicago*, respectively, as shown by unconstrained principal coordinate analysis (PCoA) using Bray–Curtis distances (*P* = 0.001; Fig. 6c). In contrast, *V. paradoxus* CL014 and *Polaromonas* MF047 had more modest effects (17.1–28.3%, *P* = 0.05), consistent with their lower colonization (Fig. 6b, Extended Data Fig. 8 and Supplementary Table 6).

Notably, no major differences were observed between wild-type and knock-in strains of *V. paradoxus* CL014, *Paraburkholderia* MF376 and *Polaromonas* MF047, suggesting that IAA degradation pathway did not markedly alter their interactions within the microbiome (Fig. 6c, Extended Data Fig. 8). Log_2_ fold-change analysis revealed no significant differences in the relative abundance of any genera between wild-type and engineered strain inoculated conditions, further suggesting that microbiome shifts were primarily driven by colonization capacity rather than IAA degradation activity (Extended Data Fig. 9).

To evaluate potential benefits for plant performance, we inoculated untreated natural soil with each strain and measured fresh shoot weight after 33 days. *Arabidopsis* grown in soil supplemented with *Paraburkholderia* MF376 carrying the complete *iad* pathway (*marR73 iadC-K2*) exhibited a significant increase in shoot biomass compared to uninoculated controls and those treated with the wild-type strain (Fig. 6d, Extended Data Fig. 10, and Supplementary Table 7). These findings indicate that combining a strong plant colonizer chassis strain with the full IAA degradation pathway can enhance plant growth in natural soil, offering a promising strategy for microbiome-based agricultural interventions.

## Discussion

Members of the genus *Variovorax* play a critical role in plant–microbe interactions by mitigating RGI induced by auxin-producing bacteria, establishing them as key plant-beneficial microbes^5,8,34,35^. Building on this foundation, we used bacterial genetics, metabolomics, and isotope tracing to dissect the *iad*-mediated IAA degradation pathway in *V. paradoxus* CL014. We illuminated the *iad* pathway biochemistry and defined the *iad* genes associated with each pathway step. We then used this knowledge to evaluate the functional transferability of the *iad* pathway to other root-associated bacteria and assess the effects of these engineered strains on microbiome composition and plant phenotype.

A previous study using [^13^C_6_]IAA (benzene ring-labeled) proposed that *V. paradoxus* CL014 degrades IAA via a pathway similar to that of *B. japonicum*, in which IAA is sequentially processed through 2-hydroxyindole-3-acetic acid, dioxindole-3-acetic acid (DOAA), isatin, isatinic acid, and anthranilic acid^9,13^. However, since key structural modifications occur at the pyrrole ring and acetic acid side chain, we used [^2^H_7_]IAA to track these changes with greater resolution. Surprisingly, our isotope tracing revealed a distinct degradation route (Fig. 2c). Rather than proceeding through the 2-hydroxyindole-3-acetic acid or 2-oxindole-3-acetic acid pathway, IAA was metabolized via a likely epoxide mechanism followed by hydration, and a subsequent dehydrogenation, ultimately leading to the production of the final product, anthranilic acid (Fig. 2c). This finding redefines the function of IadDE: although annotated as, and most structurally similar to Rieske non-heme dioxygenases (Fig. 3a, b), it functions as a monooxygenase (Fig. 2). While classical Rieske dioxygenases typically catalyze dioxygenation reactions, exceptions such as naphthalene dioxygenase have been shown to perform monooxygenation via radical intermediates^15^. However, indole oxidation has rarely been attributed to Rieske-type enzymes and is more commonly associated with the tryptophan 2,3-dioxygenase (TDO) superfamily, which features a heme cofactor coordinated by a conserved histidine and a distinct core architecture^22,36^. Despite their structural divergence, the catalytic mechanism of IadCDE parallels that of MarE, a heme-dependent monooxygenase in the TDO superfamily that catalyzes 2-oxoindole formation during maremycin biosynthesis in *Streptomyces* (Extended Data Fig. 5)^17,18^. While some TDO enzymes catalyze dioxygenation via two consecutive monooxygenation steps, others—including MarE, SfmD, and TyrH—mediate single oxygen-atom transfer, with the second oxygen likely reduced to water in the presence of an electron donor such as ascorbate^17–19,22,37^. Given the central role of oxygenases in bacterial aromatic compound degradation^14^, our findings provide new mechanistic insights into O_2_-dependent ring cleavage strategies that overcome the inherent chemical stability of aromatic substrates. This revised pathway highlights the functional diversity of oxygenases and suggests that IadDE-mediated degradation of indole compounds more closely resembles the activity of heme-dependent monooxygenases than classical Rieske-type dioxygenases, representing a distinct biochemical route for aromatic catabolism (Extended Data Fig. 5). Notably, pathways for direct conversion of indoles to oxindoles have garnered increasing attention due to their relevance to various pathogenic processes in humans and the multipotent therapeutic value of oxindole pharmacophores^16^. Our study expands the understanding of microbial oxygenase diversity and provides a valuable link between bacterial aromatic catabolism and broader biological processes involving indole monooxygenation.

IadC and IadDE form a two-component oxygenase system, with IadC transferring electrons from NADH to IadDE to activate molecular oxygen^12,26^. Although IadC enhances catalytic efficiency^9^, it is dispensable in *V. paradoxus* CL014, likely due to functional redundancy with endogenous reductases. This is supported by the observation that overexpression of *iadDE* alone in the ΔHS33 background restores IAA degradation^9^. Moreover, IadDE activity is strain-dependent when expressed heterologously; it functions in *E. coli* 10 Beta but not in BL21(DE3), likely due to host-specific differences in redox environments^9,12^. Analogous redundancy has been reported in *Sphingopyxis granuli*, where ThnA4, a ferredoxin reductase, is dispensable for tetralin degradation^38^. While *iadC* is not required for IAA degradation in *V. paradoxus* CL014, it is essential for RGI mitigation. In *Arabidopsis*, only ΔHS33 strains expressing *iadCDE*, but not *iadDE* alone, reversed RGI induced by the auxin-producing *Arthrobacter* CL028^9^, indicating that *iadC* is essential for functional rescue.

To test broader applicability, we engineered *Polaromonas* MF047 and *Paraburkholderia* MF376 with the same two versions of the *iad* pathway, selecting these chassis strains for their root-association and potential plant-beneficial traits. All engineered strains degraded IAA and alleviated RGI induced by exogenous IAA, *Arthrobacter* CL028, and SynCom32 (Fig. 4). Notably, *Paraburkholderia* MF376 consistently outperformed *Polaromonas* MF047 across all tested conditions and reversed RGI caused by *Pseudomonas* MF048, where *V. paradoxus* CL014 is ineffective (Fig. 5c). 16S rRNA amplicon sequencing revealed that integration of the *iad* pathway into any of these strains (ΔHS33, MF047, or MF376) did not substantially alter root community structure compared to the respective wild type (Fig. 6b). Instead, colonization capacity emerged as the major driver of microbiome shifts (Fig. 6b, c; Extended Data Fig. 8). Among all tested strains, *Paraburkholderia* MF376 strains exhibited the highest colonization efficiency whether wild type or engineered (Fig. 6b). And, in natural soil with a native microbiome, the engineered *Paraburkholderia* MF376 strain significantly enhanced plant growth, increasing shoot biomass relative to uninoculated controls and the wild type (Fig. 6d, Extended Data Fig. 10). This beneficial effect likely reflects its superior root colonization ability and the efficient IAA-degrading activity introduced through our engineering. Together, our findings underscore the importance of chassis strain selection when engineering IAA-degradation into new bacteria and demonstrate that combining heterologous expression of the *iad* genes with strong root colonization appears best for promoting rhizosphere auxin homeostasis and plant growth. This study advances our mechanistic understanding of microbial auxin metabolism and provides a platform for designing next-generation microbial bioinoculants to modulate plant-microbe interactions and enhance crop productivity in diverse soil environments.

## Methods

### Engineering bacterial strains

#### Knock-out mutant construction.

Gene deletions in *V. paradoxus* CL014 were generated using the suicide vector pMo130, following previously established methods^5,9,39^. The vector backbone was PCR amplified and treated with DpnI to remove the template DNA. Primers used for mutant construction are listed in Supplementary Table 2. Upstream and downstream flanking regions of target genes were amplified from *V. paradoxus* CL014 genomic DNA using Platinum SuperFi II PCR Master Mix (Thermo Fisher Scientific). PCR products were purified using the DNA Clean & Concentrator Kit (Zymo Research) and assembled into pMo130 using HiFi Gibson Assembly Master Mix (New England Biolabs). Assembled plasmids were transformed into *E. coli* NEB 5-alpha (New England Biolabs), selected on Lysogeny Broth (LB, Thermo Fisher Scientific, BP1427) agar (2% w/v) supplemented with kanamycin (50 μg/mL), and verified by Sanger sequencing (Genewiz). Sequence-confirmed plasmids were transferred into the diaminopimelic acid (DAP) auxotrophic *E. coli* WM3064 for conjugation. Transformants were selected on LB agar containing kanamycin (50 μg/mL) and DAP (0.3 mM), and grown at 37 °C for 24 h. For biparental mating, donor *E. coli* WM3064 and recipient *V. paradoxus* CL014 (pre-grown in 50% TSB (Tryptic Soy Broth, Thermo Fisher Scientific, CM0129) agar (2% w/v) with ampicillin (100 μg/mL)) were washed twice with 50% TSB, mixed at a 1:1 volume ratio, pelleted (5,000 × *g*, 5 min), resuspended in 1/10 volume of 50% TSB, and spotted onto 50% TSB agar containing DAP (0.3 mM). After overnight incubation at 28 °C, exconjugants were selected on 50% TSB agar containing ampicillin and kanamycin without DAP, and incubated for 3–4 days. Resulting colonies were re-streaked onto fresh antibiotic 50% TSB agar to ensure clonality and remove residual donor cells. Single colonies were screened by colony PCR to confirm single-crossover integration. Verified integrants were cultured in 50% TSB with ampicillin and Isopropyl β-D-1-thiogalactopyranoside (IPTG, 1 mM). Cultures were plated on sucrose counter-selection agar (10 g/L tryptone, 5 g/L yeast extract, 10% w/v sucrose, 2% w/v agar, 100 μg/mL ampicillin, 1 mM IPTG) and incubated at 28 °C for 3–4 days. Resulting colonies were passaged in the same liquid medium, and deletion events were verified by PCR. Final deletion strains were re-streaked on 50% TSB with ampicillin and confirmed by diagnostic PCR using one primer located outside the deletion region and one within the deleted gene to ensure complete excision and strain purity. DNA templates for all PCRs were prepared using the following rapid lysis protocol^40^. A single colony or 6 μl of bacterial culture was mixed with 10 μl of alkaline lysis buffer (25 mM NaOH, 0.2 mM Na_2_-EDTA, pH 12), incubated at 95 °C for 30 min, and neutralized with 10 μl Tris-HCl (40 mM, pH 7.5). The resulting material was used directly as PCR template (1:10, template:total PCR reaction volume).

#### Overexpression mutant construction.

*V. paradoxus* CL014 *marR73 iadCDE* and *marR73 iadC-K2* were cloned into the broad-host-range vector pBBR1MCS-2 as previously described^5,9,41^, genomic fragments were amplified using Platinum SuperFi II PCR Master Mix and assembled into pBBR1MCS-2 via Gibson assembly using HiFi Gibson Assembly Master Mix (New England Biolabs). Primers used for mutant construction are listed in Supplementary Table 2. Circular template DNA was digested with DpnI, and assembled plasmids were transformed into *E. coli* NEB 10-beta (New England Biolabs). Transformants were selected on LB agar containing kanamycin (50 μg/mL), and plasmids were extracted (ZR Plasmid Miniprep Kit, Zymo Research) and verified by Sanger sequencing. Verified constructs were introduced into *V. paradoxus* CL014 ΔHS33 by tri-parental mating, using *E. coli* pRK2013 as a helper strain. Donor and helper strains were grown in LB with kanamycin (50 μg/mL) at 37 °C, and the recipient strain was cultured in 50% TSB with ampicillin (100 μg/mL) at 28 °C. All strains were pelleted (5,000 × *g*, 5 min), washed in 50% TSB twice, mixed at equal volumes, and spotted onto 50% TSB agar for overnight conjugation at 28 °C. Exconjugants were selected on 50% TSB agar supplemented with kanamycin (50 μg/mL) and ampicillin (100 μg/mL), confirming successful plasmid transfer into *V. paradoxus* CL014 ΔHS33.

#### Knock-in mutant construction.

Two gene combinations, *marR73 iadCDE* and *marR73 iadC-K2*, were integrated into the *iad* locus of *V. paradoxus* CL014 ΔHS33, as well as three intergenic genomic sites in *Polaromonas* MF047 (IMG genome ID 2636416056 with insertion positions between Gene IDs: 2639079279–80, 2639079819–20, and 2639080354–55) and one site in *Paraburkholderia* MF376 (IMG genome ID 2521172625 with insertion position between Gene IDs 2521671121–22), using the pMo130 suicide vector (Extended Data Fig. 6b). Gene amplification, vector assembly, and verification followed the same procedure as knockout mutant construction, except that plasmids were initially propagated in *E. coli* NEB 10-beta. Primers used for mutant construction are listed in Supplementary Table 2. Sequence-verified plasmids were electroporated into *V. paradoxus* CL014 ΔHS33, *Polaromonas* MF047, and *Paraburkholderia* MF376, as described below. Strains were cultured in 50% TSB at 28 °C with shaking (250 rpm) for 2 days, followed by 24 h incubation at 4 °C. Cells were harvested (5,000 × *g*, 10 min, 4 °C), washed twice with ice-cold sterile water, and resuspended in 10% sterile glycerol for electroporation. For each reaction, 100 ng of plasmid DNA was electroporated into 100 μl of competent cells using a 0.1 cm gap cuvette using the following conditions: 1,800 V, 25 μF, 200 Ω for *marR73 iadCDE*, and 2,500 V, 25 μF, 200 Ω for *marR73 iadC-K2*. After electroporation, cells were recovered in SOC medium (New England Biolabs, B9020) at 28 °C (250 rpm) for 3 h and plated on 50% TSB agar with selective antibiotics: ampicillin (100 μg/mL) + kanamycin (200 μg/mL) for *Polaromonas* MF047 and kanamycin (50 μg/mL) for *Paraburkholderia* MF376. After 4–5 days of incubation, single colonies were screened by colony PCR using crude DNA extracted with alkaline lysis buffer. PCR-confirmed integrants were grown overnight in 50% TSB with 1 mM IPTG, supplemented with ampicillin (100 μg/mL) for *Polaromonas* MF047 and without antibiotics for *Paraburkholderia* MF376. Cultures were then diluted 1,000-fold and plated on sucrose counter-selection agar to induce second recombination (5% w/v sucrose for *Polaromonas* MF047; 20% w/v sucrose for *Paraburkholderia* MF376). Double-crossover mutants were confirmed by PCR and Sanger sequencing.

### Liquid chromatography -mass spectroscopy (LC–MS) metabolomics

#### Sample preparation.

*V. paradoxus* strains were streaked from glycerol stocks onto 50% TSB agar plates supplemented with appropriate antibiotics: ampicillin (100 μg/mL) for knockout mutants and wildtype, and ampicillin (100 μg/mL) plus kanamycin (50 μg/mL) for overexpression mutants. Plates were incubated at 28 °C for 3 days. Single colonies were inoculated into 5 mL of 50% TSB containing the appropriate antibiotics and cultured at 28 °C with shaking at 250 rpm for 48 h. Bacterial cultures were harvested and washed following the root growth inhibition assay protocol, then resuspended in 5 mL of modified M9 medium supplemented with 15 mM succinic acid to a final OD_600_ of 0.05. Cultures were incubated at 28 °C, 250 rpm for 15 h, after which IAA or deuterium-labeled IAA ([^2^H_7_]IAA, DLM-8040–0.1, Cambridge Isotope Laboratories) was added to a final concentration of 0.1 mg/mL. Cultures were incubated for an additional 4 h. Cells were collected at a total biomass of OD_600_ × volume (mL) = 2, centrifuged at 5,000 × *g* for 10 min, and pellets were resuspended in 400 μl of cold quenching solvent (acetonitrile : methanol : water, 40:40:20, v/v/v). Samples were stored at −80 °C prior to metabolite extraction and LC–MS/MS analysis.

#### LC-MS analysis.

LC-MS analysis was performed on a Vanquish UHPLC system (Thermo Fisher Scientific) coupled to a quadrupole Orbitrap Exploris 480 mass spectrometer. LC separation of polar metabolites was achieved using a Waters XBridge BEH Amide column (2.1 mm × 150 mm, 2.5-μm particle size, 130-Å pore size). The LC method has a 25-min solvent gradient at a flow rate of 150 μL/min, with the following gradient parameters: 0 min, 90% B; 2 min, 90% B; 3 min, 75%; 7 min, 75% B; 8 min, 70%, 9 min, 70% B; 10 min, 50% B; 12 min, 50% B; 13 min, 25% B; 14 min, 25% B; 16 min, 0% B, 20.5 min, 0% B; 21 min, 90% B; 25 min, 90% B, where Solvent A was 95:5 water : acetonitrile with 20 mM ammonium hydroxide and 20 mM ammonium acetate (pH 9.4) and solvent B was acetonitrile. The autosampler temperature was 4 °C, the column temperature was 25 °C, and the injection volume was 10 μl. The Exploris 480 mass spectrometer was operated in full scan mode in negative polarity on MS1 level, which allows the relative quantitation of the metabolite across by ion count. Following parameters are used for the full scan: resolution, 120,000; scan range, m/z 70–1000 (negative mode); AGC target, 1e6; ITmax, 500 ms. Other instrument parameters are spray voltage 3000 V, sheath gas 35 (Arb), aux gas 10 (Arb), sweep gas 0.5 (Arb), ion transfer tube temperature 300 °C, vaporizer temperature 35°C, internal mass calibration on, RF lens 50. The MS2 spectra were collected in targeted mode using the parallel reaction monitoring (PRM) function at higher energy C-trap dissociation (HCD) energy of 20eV, and other instrument settings as following: resolution 30,000, AGC target 1e6, maximum injection time 250 ms, and isolation window 1.0 m/z. For the MS1 data analysis, raw LC–MS data were converted to mzXML format using *ProteoWizard*^42^ Peak picking was performed with EL-Maven (v0.12.1-beta; Elucidata) for unlabeled and ^2^H-labeled compounds, and MAVEN (v2.10.14c) for ^18^O-labeled compounds. Relative abundance changes of each metabolite were quantified using relative peak area tops in the chromatogram. For ^2^H-labeled data analysis, natural isotope abundance was corrected using the AccuCor R package^43^ (https://github.com/lparsons/accucor). For ^18^O-labeled data analysis, isotope correction was performed using the Iso-Autocorr package (https://github.com/xxing9703/Iso-Autocorr). For the MS2 data, raw LC–MS files were processed and peaks were extracted with the built-in Xcalibur Qual Browser (Thermo Scientific, v4.4).

### IadDE protein expression, purification and crystallization

#### Protein expression.

The *iadD* and *iadE* genes (IMG gene IDs: 2643613669, 2643613668) from *V. paradoxus* CL014 (IMG genome ID: 2643221508) were cloned into the pET28b expression vector with an N-terminal His-tag and transformed into *E. coli* BL21(DE3) for recombinant protein expression. Transformants were plated on LB agar supplemented with kanamycin (50 μg/mL) and chloramphenicol (33 μg/mL) and incubated at 37 °C for 24 h. Individual colonies were picked and grown overnight in LB medium at 37 °C with shaking at 250 rpm. Overnight cultures were diluted 1:1000 into autoinduction (AI) medium (ZYM-5052)^44^ containing the same antibiotics, and incubated at 37 °C, 250 rpm for 22 h. Cells were harvested by centrifugation at 5,000 × *g* for 20 min at 4 °C, and pellets were collected and stored at −80 °C for subsequent protein purification.

#### Protein purification.

Cell pellets were resuspended in IMAC Buffer A (20 mM NaH_2_PO_4_, 500 mM NaCl, pH 7.4) at a ratio of 1 g pellet per 10 mL buffer. To reduce viscosity, 0.1 μL benzonase nuclease (Sigma-Aldrich, 70746) was added per gram of cell pellet. Cells were lysed using an EmulsiFlex-C5 high-pressure homogenizer (three passes), and the lysate was clarified by centrifugation at 25,000 × *g* for 30 min at 4 °C. The supernatant was filtered through a 0.22 μm syringe filter and loaded onto a 5 mL Ni-charged Nuvia IMAC column (Bio-Rad) using a fast protein liquid chromatography (FPLC) system (Bio-Rad). The column was washed with IMAC Buffer A containing 15 mM imidazole, and bound proteins were eluted with IMAC Buffer B (20 mM NaH_2_PO_4_, 500 mM NaCl, 500 mM imidazole, pH 7.4). Eluted fractions were analyzed by SDS–PAGE (Bio-Rad Stain-Free gels), pooled, concentrated, and buffer-exchanged into 50 mM Tris-HCl (pH 7.0), 150 mM NaCl using 10 kDa MWCO concentrators (Pierce, Thermo Fisher). Purified protein was stored at 4 °C, and concentration was determined using the Pierce BCA Protein Assay Kit (Thermo Fisher Scientific).

#### Protein crystallography.

Purified IadDE was concentrated to 10 mg/mL in buffer supplemented with 1 mM dithiothreitol (DTT) and crystallized using the sitting-drop vapor diffusion method at 20 °C. Crystallization drops were prepared by mixing 1.5 μl of protein solution with 1.5 μl of reservoir solution in 24-well sitting-drop plates. The reservoir solution contained 9.6–10.6% (w/v) PEG 3350 and 0.1 M sodium citrate tribasic dihydrate (pH 5.5). Crystals were cryoprotected by brief soaking in the reservoir solution supplemented with 30% (v/v) ethylene glycol and subsequently flash-cooled in liquid nitrogen for data collection. Diffraction data were collected at beamlines 17-ID1 (AMX) and 17-ID2 (FMX) at Brookhaven National Laboratory to a maximum resolution of 1.28 Å. Crystals grew in space group H3 (hexagonal setting of R3) with typical cell dimensions a=b=130.5 Å c=100.7 Å α=β=90° γ=120° with one complex per asymmetric unit. Data were processed with XDS^45^ and scaled with AIMLESS^46^. The structure was determined by the method of molecular replacement using the program PHASER^47^ utilizing sequential placement of the two subunits with the models derived from AlphaFold models. The structure was rebuilt in COOT^48^, incorporating a [2Fe–2S] cluster and a mononuclear iron-binding site, and subsequently refined using PHENIX.REFINE^49^. The final model showed good agreement with the experimental data and displayed excellent geometry. The final model and associated X-ray data have been deposited with the Protein Data Bank with code 9O71. Relevant refinement statistics are summarized in Supplementary Table 5.

### Measurement of IAA degradation

IAA degradation by bacterial strains was assessed using a spike-in approach. Bacterial preparation followed the same procedure as the root growth inhibition assay, including streaking from glycerol stocks, cultivation, washing, and OD_600_ measurement. Washed cells were inoculated into M9 medium^5,9^ (3 g/L KH_2_PO_4_, 0.5 g/L NaCl, 6.78 g/L Na_2_HPO_4_, and 1 g/L NH_4_Cl), supplemented with 2 mM MgSO_4_, 0.1 mM CaCl_2_, 10 μM FeSO_4_, and 5 g/L glucose, to a final OD_600_ of 0.05. Cultures were incubated at 28 °C with shaking (250 rpm) for 15 h before IAA was spiked-in to a final concentration of 0.1 mg/mL. Aliquots (300 μl) were collected at 2 h or 4 h intervals, centrifuged at 5,000 × *g* for 10 min, and 50 μl of the supernatant was mixed with 100 μl of freshly prepared Salkowski reagent (10 mM FeCl_3_ and 35% perchloric acid). After incubation for 40 min at room temperature, absorbance was measured at 530 nm using a BioTek Synergy H1 microplate reader. As *Polaromonas* MF047 is an amino acid auxotroph and cannot grow in minimal medium, this strain and its engineered strains were assayed in 50% TSB medium instead of M9. All strains were tested in three biological replicates to ensure reproducibility.

### Bacterial growth curve

Bacterial preparation followed the same protocol as the root growth inhibition assay. Washed cells were inoculated into 200 μl of either M9 minimal medium or 50% TSB medium at a final OD_600_ of 0.05. Each strain was tested in three biological replicates to ensure reproducibility. Cultures were grown in sterile 96-well cell culture plates, sealed with Breathe-Easy gas-permeable film (Diversified Biotech), and incubated at 28 °C with continuous linear shaking. Optical density at 600 nm (OD_600_) was recorded every 2 h over a 24-hour period using a BioTek Synergy H1 microplate reader to monitor bacterial growth dynamics.

### Root growth inhibition assay

#### Seedling and seed preparation.

*Arabidopsis thaliana* Col-0 seeds were surface-sterilized by vortexing in 70% bleach containing 0.2% Tween-20 for 10 min, followed by five washes with sterile distilled water. Seeds were sown on half-strength Murashige and Skoog (MS) agar medium (2.22 g/L MS basal medium with Gamborg vitamins (PhytoTech Labs, M-404), 0.5 g/L MES, 5 g/L sucrose, 10 g/L agar, pH 5.7 adjusted with 3 M NaOH) in 12 × 12 cm square plates and grown vertically under short-day conditions (21 °C day / 18 °C night, 10 h light / 14 h dark, 70% relative humidity, 170 μmol m^−2^ s^−1^ light intensity) for 7 days. *Medicago* seeds were sterilized following published protocols^50,51^. Briefly, seeds were treated with concentrated sulfuric acid for 10 min with agitation, rinsed once with sterile water, then treated with 70% bleach for 3 min. After five additional washes, seeds were imbibed in sterile water for 2–6 h at room temperature prior to use.

#### IAA-containing plate preparation.

Half-strength MS agar medium was autoclaved and cooled until warm to the touch. IAA stock solutions (1 mM or 100 mM in 100% ethanol) were added to final concentrations of 100 nM, 1 μM, or 10 μM. Stocks were stored at – 20 °C for up to two months.

#### Bacterial and SynCom32 preparation.

Bacterial strains were revived from 20% glycerol stocks by streaking onto 50% TSB agar plates (15 g/L tryptic soy broth, 20 g/L agar) and incubated at 28 °C for 3–4 days. Single colonies were inoculated into 5 mL 50% TSB and grown for 2 days at 28 °C with shaking (250 rpm). Cultures were pelleted at 5,000 × g for 10 min, washed twice with 3 mL of sterile 10 mM MgCl_2_, and resuspended in 750 μl of the same buffer. The optical density at 600 nm (OD_600_) was measured using a NanoDrop One C with semi-micro cuvettes and adjusted to 0.05. For SynCom32 assembly, each strain’s OD_600_ was measured individually. To ensure equal biomass contribution, volumes were calculated using the equation: OD_600_ × volume (μl) = 300, and combined accordingly. The pooled culture was washed and resuspended as described above.

#### Bacterial inoculation.

For plant-microbe co-inoculation assays, 100 μl of strain (OD_600_ = 0.05) was evenly spread onto the surface of half-strength MS agar plates. For SynCom32, 100 μl of the pooled culture (OD_600_ = 0.05) was applied, followed by 100 μl of *V. paradoxus* CL014, *Polaromonas* MF047, *Paraburkholderia* MF376, or their engineered strains at OD_600_ = 0.005. Plates were incubated overnight at room temperature prior to seedling transfer.

#### Plant growth and measurement.

Seven-day-old *Arabidopsis* seedlings or sterilized *Medicago* seeds were transferred onto pre-inoculated plates. Plates were sealed with 3M Micropore tape and incubated vertically in a growth chamber under short-day conditions for 7 days. For 16S rRNA amplicon sequencing, plants were grown for 9 days to allow sufficient biomass for DNA extraction. Plates were imaged using a digital camera, and primary root elongation was measured as the distance from the initial to final root tip position using the freehand line tool in ImageJ. All primary root elongation data are provided in Supplementary Table 3.

### Microbiome analysis

#### Sample collection.

After nine days of co-incubation with bacteria, roots from *Arabidopsis* and *Medicago* were harvested for microbiome profiling. Each sample consisted of 5–10 roots pooled per plate, with 3–5 biological replicates per treatment. Roots were transferred to 15 mL Falcon tubes containing 7 mL sterile water and washed by vigorous vortexing. Excess water was removed using sterile filter paper, and dried roots were transferred to Lysing Matrix E tubes (MP Biomedicals) and stored at −80 °C until DNA extraction. For SynCom32 input controls, bacterial cultures were pelleted at 5,000 × *g* for 10 min, the supernatant was discarded, and pellets were stored at −80 °C.

#### DNA extraction.

Samples were homogenized using a FastPrep-24^™^ 5G instrument (MP Biomedicals) with two 40 s cycles at 6.0 m/s for root samples, and one cycle for SynCom32 input controls, with 2 min on ice between runs to prevent overheating. DNA was extracted using the FastDNA SPIN Kit for Soil (MP Biomedicals), eluted in 55 μl DES buffer (provided in the kit), quantified using the Quant-iT PicoGreen dsDNA Assay Kit (Thermo Fisher), and diluted to 3.5 ng/μl with DES buffer.

### 16S rRNA library preparation and sequencing.

The V5–V7 region^52^ of the 16S rRNA gene was amplified using a two-step dual-indexed PCR approach. The first PCR was performed using Platinum SuperFi II PCR Master Mix (Thermo Fisher) in a 25 μl reaction containing: 12.5 μl Master Mix, 7 μl nuclease-free water (Qiagen), 2 μl of 5 μM forward primer 799F (5′-AACMGGATTAGATACCCKG-3′, with 10-bp sample barcode), 1 μl of 10 μM reverse primer 1192R (5′-ACGTCATCCCCACCTTCC-3′, with 6-bp library barcode), and 2.5 μl of 3.5 ng/μl DNA template. Thermal cycling conditions were: 98 °C for 30 s, followed by 30 cycles of 98 °C for 10 s, 55 °C for 10 s, and 72 °C for 15 s, with a final extension at 72 °C for 5 min. Three technical PCR replicates were pooled per sample to minimize amplification bias. Products were verified by 1.2% agarose gel electrophoresis. For gel purification, 25 μl of each PCR product (two samples per lane) were pooled with 10 μl 6× loading dye, run on a 1.2% agarose gel, and ~400 bp bands were excised and purified using the Wizard SV Gel and PCR Clean-Up System (Promega). DNA concentration was assessed by PicoGreen, and 100 ng of each sample was pooled for library construction and cleaned with 0.9× AMPure XP beads (Beckman Coulter). A second PCR was performed to add Illumina sequencing adapters. Final libraries were sequenced on an Illumina MiSeq platform (2 × 300 bp paired-end reads)^52,53^ at the Princeton Genomics Core Facility. After quality filtering, a total of 14,907,735 high-quality sequences were obtained from 175 samples, with an average of 85,187 reads per sample.

#### Amplicon data processing.

Raw reads were demultiplexed in QIIME2 (v2024.10)^54^ using qiime cutadapt demux-paired^55^, and primer sequences were trimmed with qiime cutadapt trim-paired. Denoising, quality filtering, and chimera removal were performed with DADA2 (qiime dada2 denoise-paired)^56^. After testing multiple truncation lengths, forward reads were truncated at 220 bp and reverse reads at 180 bp to optimize read retention and ASV diversity. ASVs were taxonomically classified using a naïve bayes classifier trained on a custom database of root-associated bacterial sequences^57^ via qiime feature-classifier classify-sklearn. Assignments were filtered based on confidence scores, prevalence, and relative abundance. Relative abundance tables were generated using qiime feature-table relative-frequency and merged with taxonomy and metadata for visualization in R using ggplot2. Beta diversity was calculated using Bray–Curtis dissimilarity (R package vegan, vegdist)^58^, followed by principal coordinate analysis (PCoA; cmdscale). Differences in community composition were assessed by PERMANOVA using adonis2^58^. Differential abundance analysis was conducted using the Mann–Whitney U test with FDR correction. Log_2_ fold changes were calculated, and heatmaps were visualized using pheatmap (R v4.4.2)^59^.

### Plant growth promotion assay in natural soil

Soil was collected from the Stony Ford Research Station (Princeton, NJ, USA), where no chemical fertilizers, pesticides, or plants had been applied or grown in recent years. The soil was sieved twice to remove rocks and plant debris, then distributed into pots placed within 9 × 13-inch aluminum foil trays. Pots were saturated overnight with 1.2 L of sterile water containing bacterial inoculum. Bacterial preparation followed the same protocol as the root growth inhibition assay, including streaking, cultivation, washing, and OD_600_ measurement. Individual bacterial strains were suspended in 1.2 L of sterile water to a final OD_600_ of 0.03 prior to soil application. Each treatment included four biological replicates (pots). *Arabidopsis* Col-0 seeds were surface sterilized as described above and placed directly onto the surface of inoculated soil. Pots were maintained under short-day conditions in a growth chamber and watered with 600–800 mL of distilled water every 4 days. After 33 days, above-ground tissues were harvested and shoot fresh weight was measured using an analytical balance.

## Supplementary Material

Supplement 1

Supplement 2

## Data Availability

All data supporting the findings of this study are available within the paper and its Supplementary Information. Source data underlying the figures are also provided with this paper. The IadDE protein structure has been deposited in the Protein Data Bank under accession code 9O71.
