# Supplementary material for: Engineering auxin degradation into root-associated bacteria promotes plant growth
Source: bioRxiv. 2025 Oct 26:2025.10.25.684584. Preprint. [Version 1] doi: 10.1101/2025.10.25.684584 (PMC12633357; doi:10.1101/2025.10.25.684584)
Supplement: Supplement 1 [file media-1.docx]

**Extended Data Figures for:**

**Engineering auxin degradation into root-associated bacteria promotes plant growth**

Ting Jiang^1, 10^, Yihui Shen^2, 3, 9, 10,^*, Xi Li^2, 3^, Michal J. Kozlowski^1^, Philip D. Jeffrey^4^, John T. Groves^2^, Joshua D. Rabinowitz^2, 3, 5^ and Jonathan M. Conway^1, 4, 6, 7, 8,^*

^1^Department of Chemical and Biological Engineering, Princeton University, Princeton, NJ, USA.

^2^Department of Chemistry, Princeton University, Princeton, NJ, USA.

^3^Lewis-Sigler Institute for Integrative Genomics, Princeton University, Princeton, NJ, USA.

^4^Department of Molecular Biology, Princeton University, Princeton, NJ, USA.

^5^Ludwig Institute for Cancer Research, Princeton Branch, Princeton, NJ, USA.

^6^Omenn-Darling Bioengineering Institute, Princeton University, Princeton, NJ, USA.

^7^High Meadows Environmental Institute, Princeton University, Princeton, NJ, USA.

^8^Andlinger Center for Energy and the Environment, Princeton University, Princeton, NJ, USA.

^9^Present address: Department of Bioengineering, University of Pennsylvania, Philadelphia, PA, USA.

^10^These authors contributed equally: Ting Jiang, Yihui Shen

* Correspondence to: yihuis@seas.upenn.edu; jmconway@princeton.edu

**
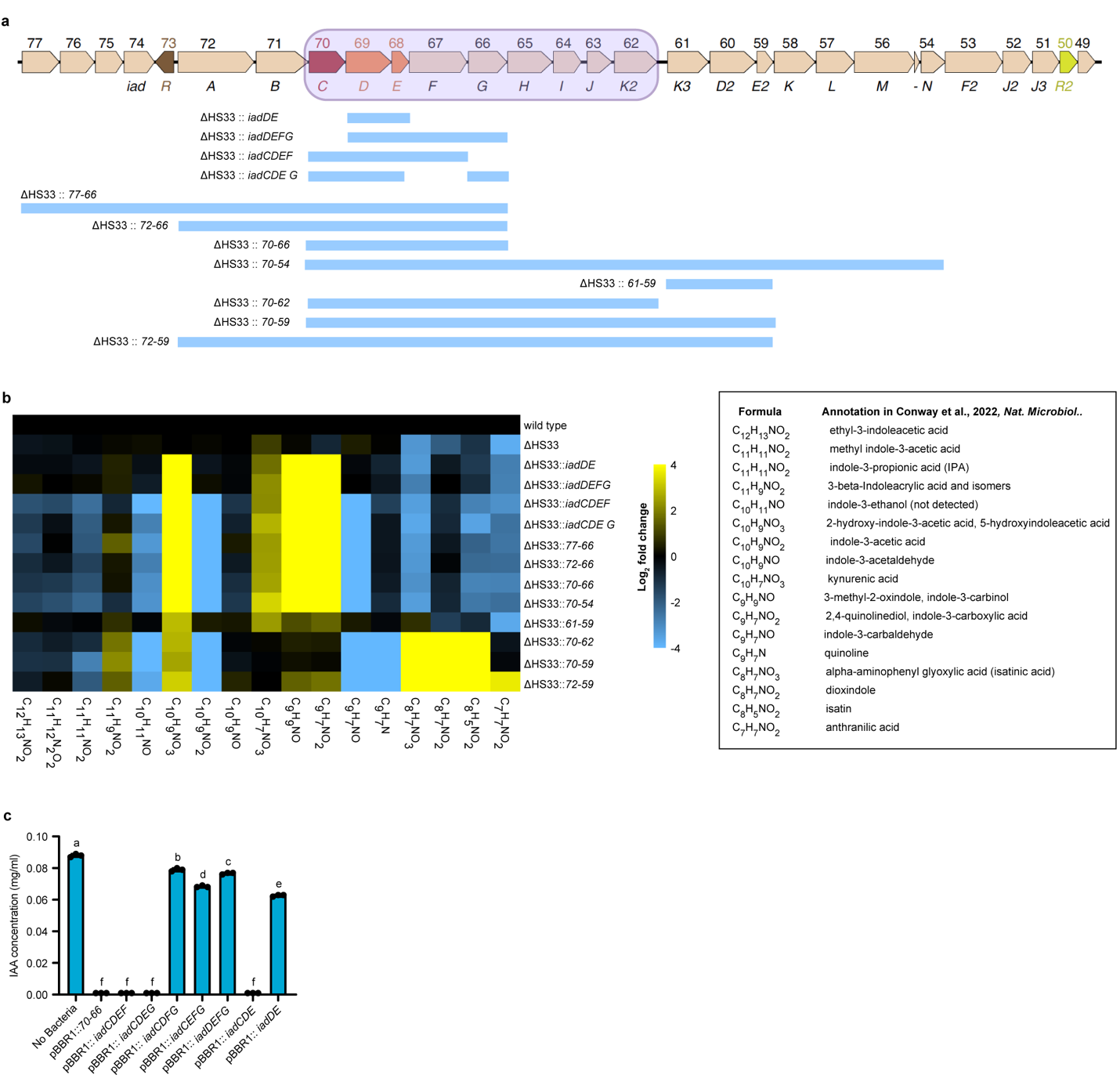
Extended Data Fig. 1 | The *iadC-K2* gene region mediates IAA degradation in *V. paradoxus* CL014. a**, Cloned fragments from the *iad* locus genomic region inserted into the broad-host-range vector pBBR1 for functional validation in Hot Spot 33 (HS33) deletion strain *V. paradoxus* ΔHS33 from^5^. **b**, Heatmap showing log₂ fold changes in metabolite abundance in complemented mutant strains relative to the wild type. Mass features (molecular formula and retention time in minutes) were selected based on previously reported IAA degradation intermediates^9^. **c**, Quantification of IAA concentration after 4 h of incubation in M9 minimal medium supplemented with 0.1 mg/mL IAA. Data represent the mean ± s.d. of *n* = 3 biological replicates. Statistical significance was assessed by one-way ANOVA followed by Tukey’s post hoc test; different letters denote statistically distinct groups.

**
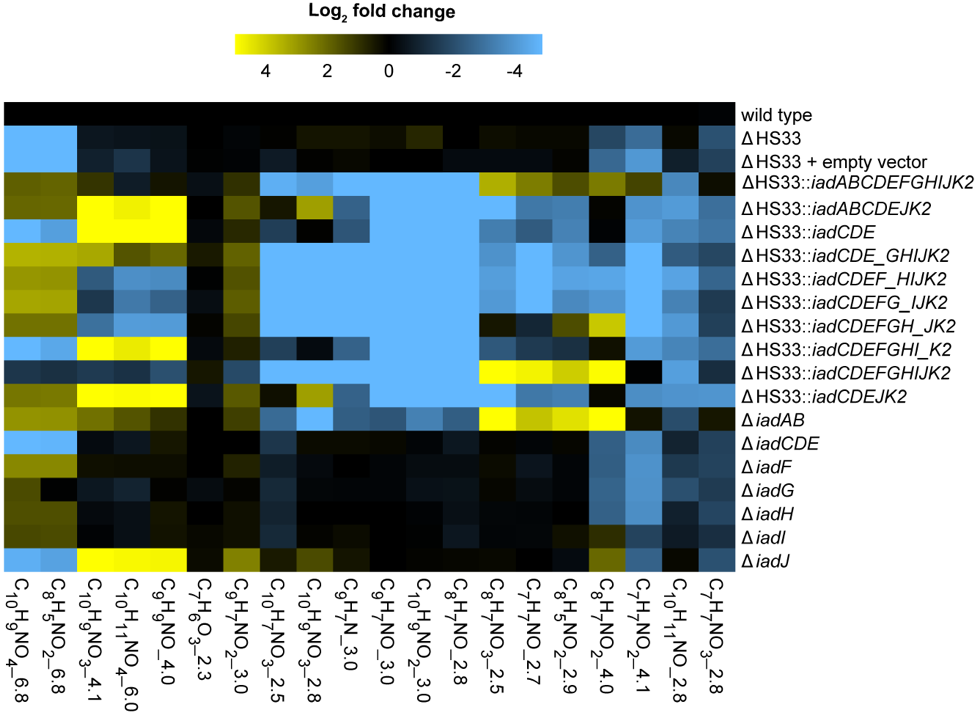
**

**Extended Data Fig. 2 | LC–MS analysis of *V. paradoxus* CL014 *iad* pathway mutants.** Each detected mass feature is annotated with its molecular formula and retention time (min). The heatmap displays log₂ fold changes in metabolite abundance relative to wild-type CL014. This dataset supports metabolite assignments and pathway inference presented in Fig. 1b. Data represent the mean of *n* = 3 biological replicates.

**
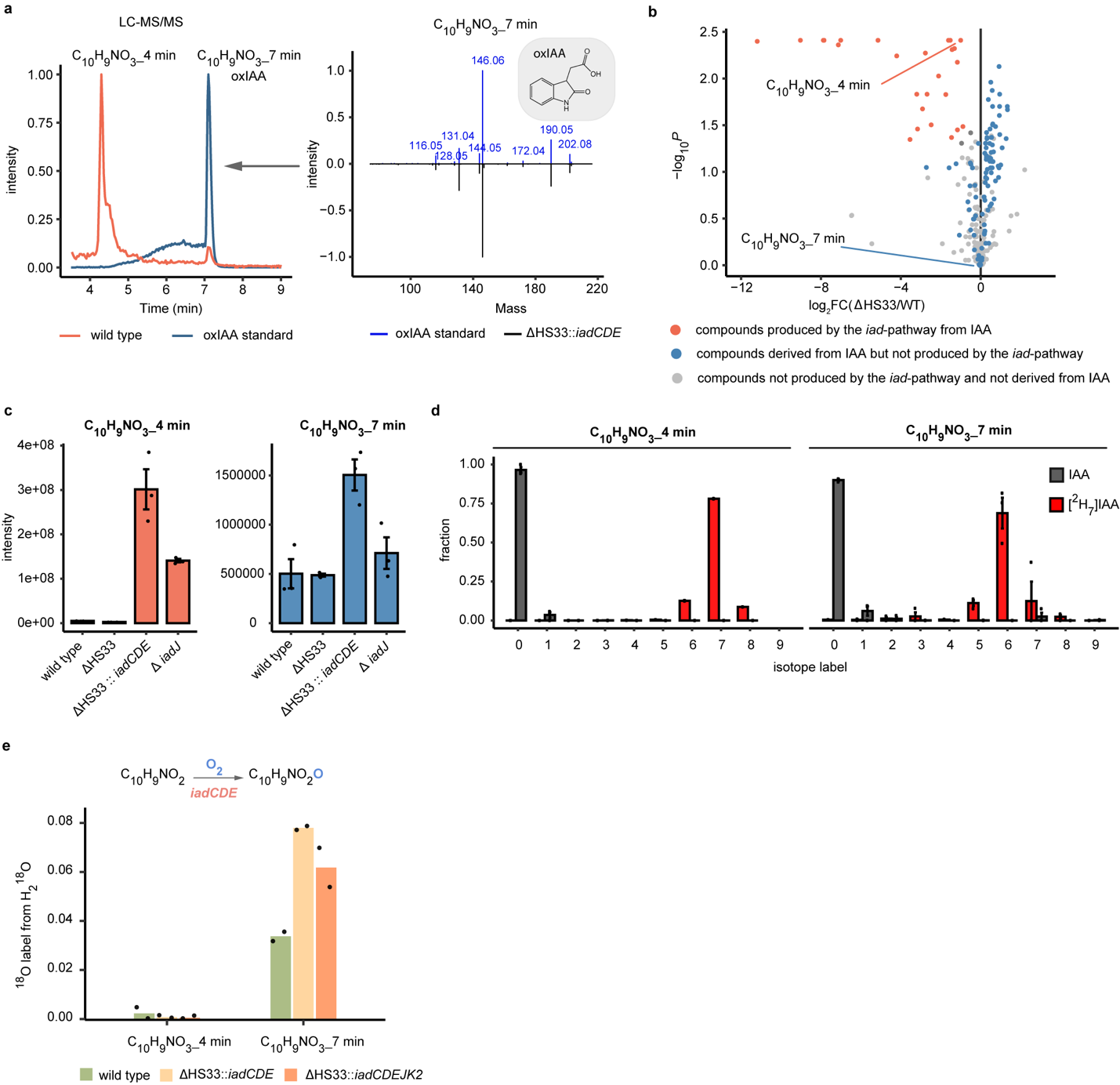
**

**Extended Data Fig. 3 | Discovery of a novel C₁₀H₉NO₃ compound distinct from oxIAA produced by IadCDE. a**, LC–MS/MS analysis identified two distinct C₁₀H₉NO₃ compounds; comparison with a standard confirmed the 7 min peak as oxIAA. **b**, Log₂ fold changes in metabolite abundance in ΔHS33 relative to wild type. Statistical significance was assessed using ANOVA followed by Benjamini–Hochberg correction for multiple comparisons. **c**, Relative abundance of the two C₁₀H₉NO₃ compounds in wild-type and mutant strains. Data represent the mean of *n* = 3 biological replicates. **d**, Deuterium isotope distribution profiles of C₁₀H₉NO₃ compounds following incubation with unlabeled IAA or [²H₇]IAA. Values represent mean ± s.e.m. of *n* = 3 biological replicates. **e**, Incorporation of ¹⁸O from H₂¹⁸O (20% v/v) into C₁₀H₉NO₃ compounds assessed by LC–MS. Data represent *n* = 2 biological replicates.

**
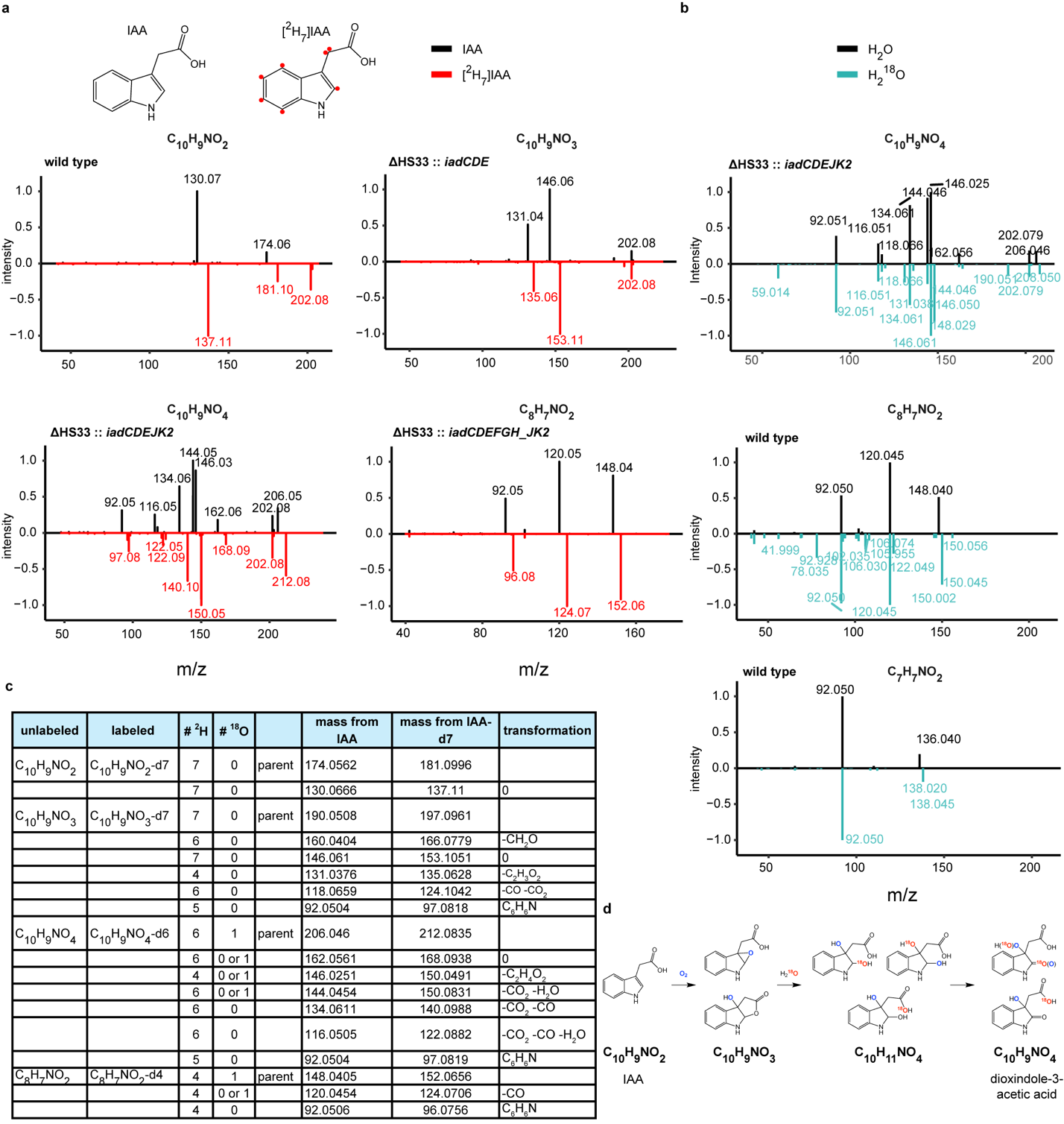
Extended Data Fig. 4 | Isotope tracing and LC-MS/MS analyses reveal atom-level transformations in IAA degradation pathway intermediates. a**, MS/MS spectra of major pathway intermediates following incubation with IAA or [²H₇]IAA. **b**, MS/MS spectra of major pathway intermediates with H₂O or H₂¹⁸O (20%, v/v). **c**, Summary table of parent ion masses, retained deuterium and ¹⁸O atoms, and major mass fragment transformations across detected intermediates. **d**, Proposed chemical structures of two-step oxidation products and their transformations, consistent with observed mass shifts and isotope retention patterns.

**
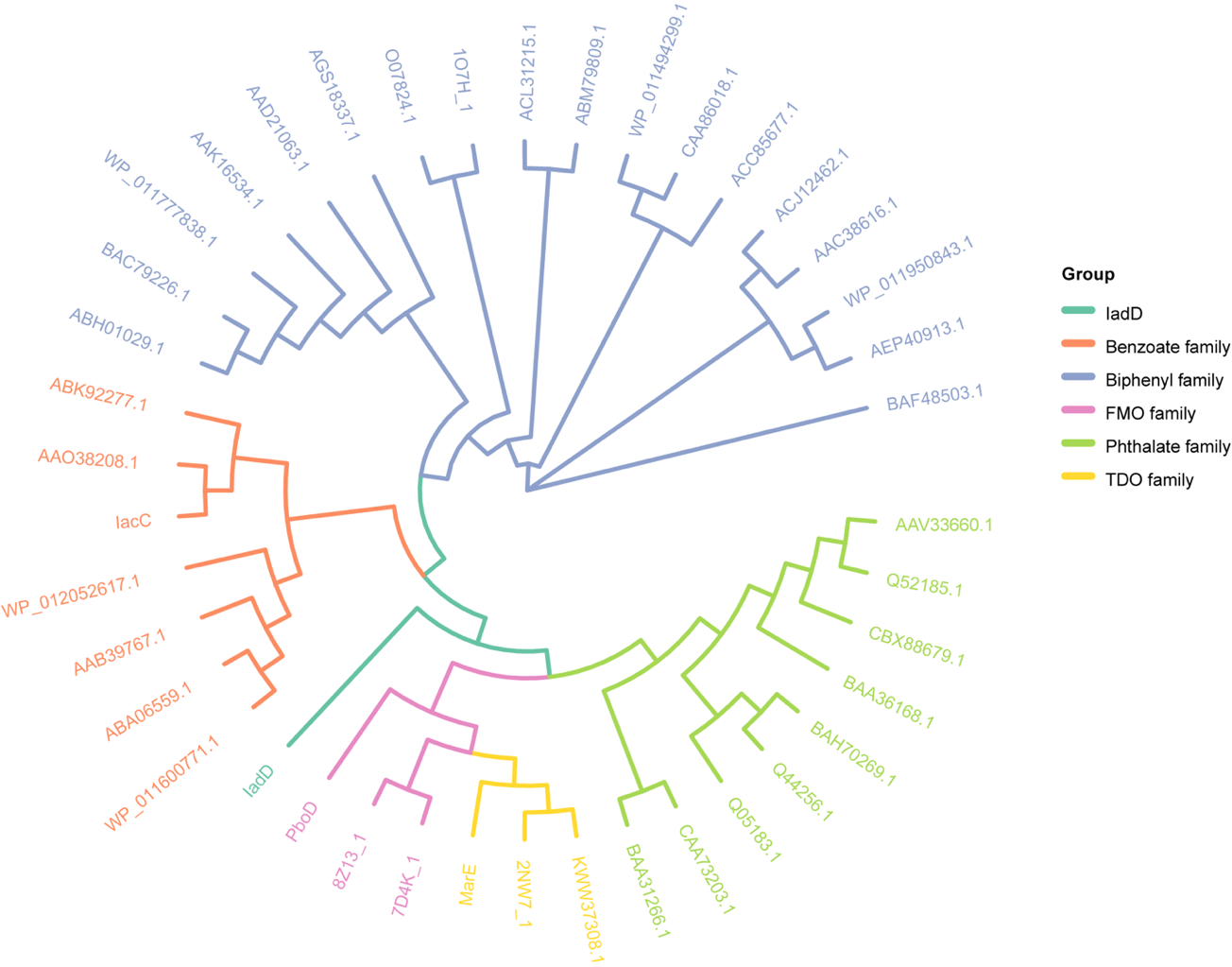
**

**Extended Data Fig. 5 | Phylogenetic analysis of IadD with Rieske dioxygenases and other indole oxygenases.** Protein sequences of characterized Rieske non-heme dioxygenases, tryptophan dioxygenases (TDOs), and flavin-dependent monooxygenases (FMOs) were obtained from NCBI and PDB (see Supplementary Table 8). Clustal Omega was used for alignment and phylogenetic tree construction. IadD from *V. paradoxus* CL014 forms a distinct subclade closely related to the phthalate dioxygenase group as well as TDO and FMO families, suggesting shared features among functionally diverse oxygenases.


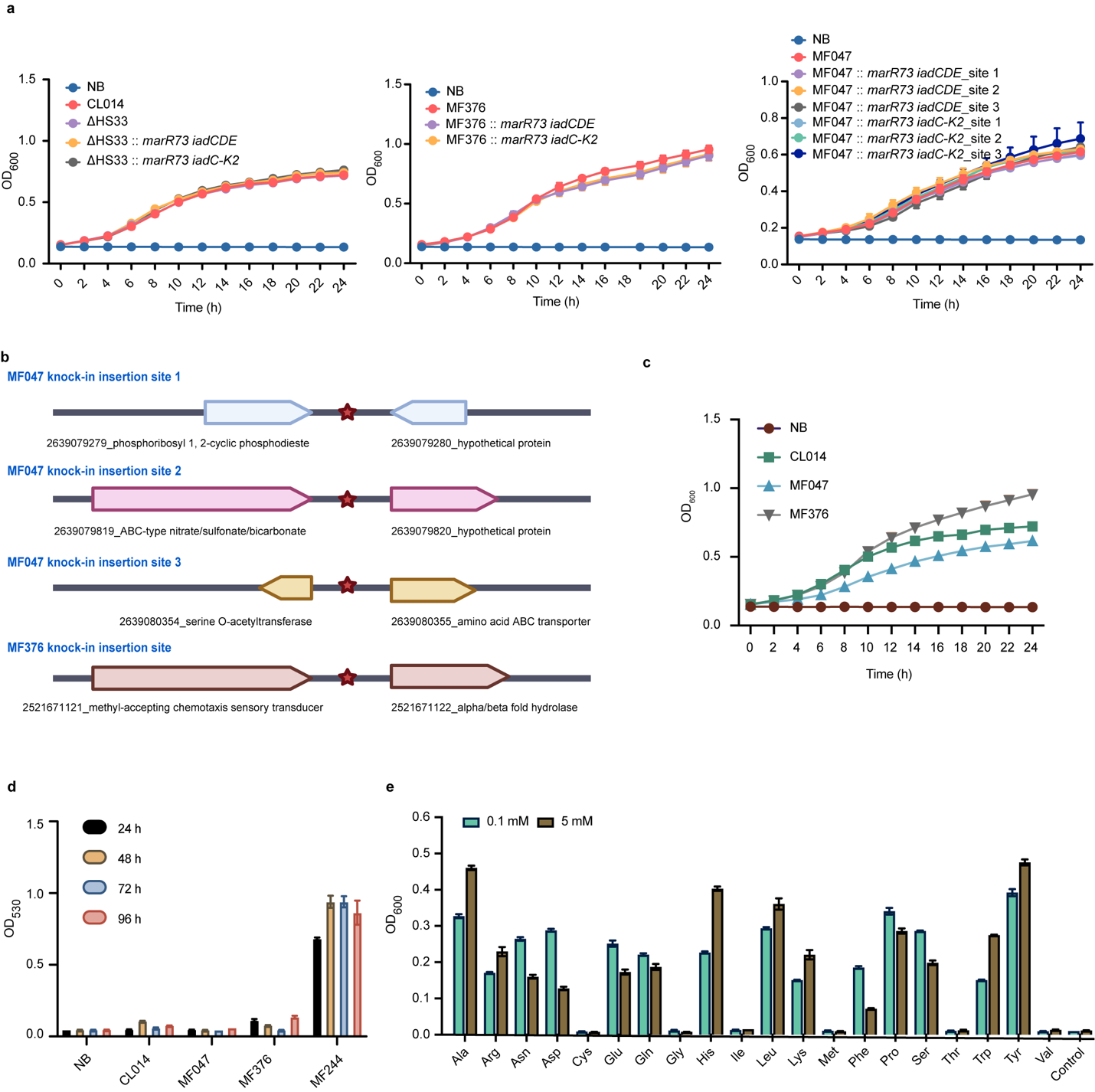
**Extended Data Fig. 6 | Characterization of candidate strains for knock-in engineering, including growth profiles, integration sites, and amino acid utilization. a,** Growth curves of engineered *V. paradoxus* CL014, *Paraburkholderia* MF376, and *Polaromonas* MF047 in 50% TSB at 28 °C. OD₆₀₀ measured over time; data are mean ± s.d. (*n* = 3). **b**, Genomic insertion sites of *iad* constructs in MF047 (3 loci) and MF376 (1 locus), based on IMG annotations. Red stars mark insertion sites. **c**, Growth curves of wild-type CL014, MF047 and MF376 under the same conditions as in (**a**). **d**, IAA production quantified by Salkowski assay in strains grown in M9 + glucose (5 g/L) + tryptophan (5 mM). *Agrobacterium* MF224 served as a positive control. **e**, Amino acid auxotrophy of MF047 assessed by growth in M9 + glucose + single amino acids (0.1 or 5 mM). Data are mean ± s.d. (*n* = 2).

**
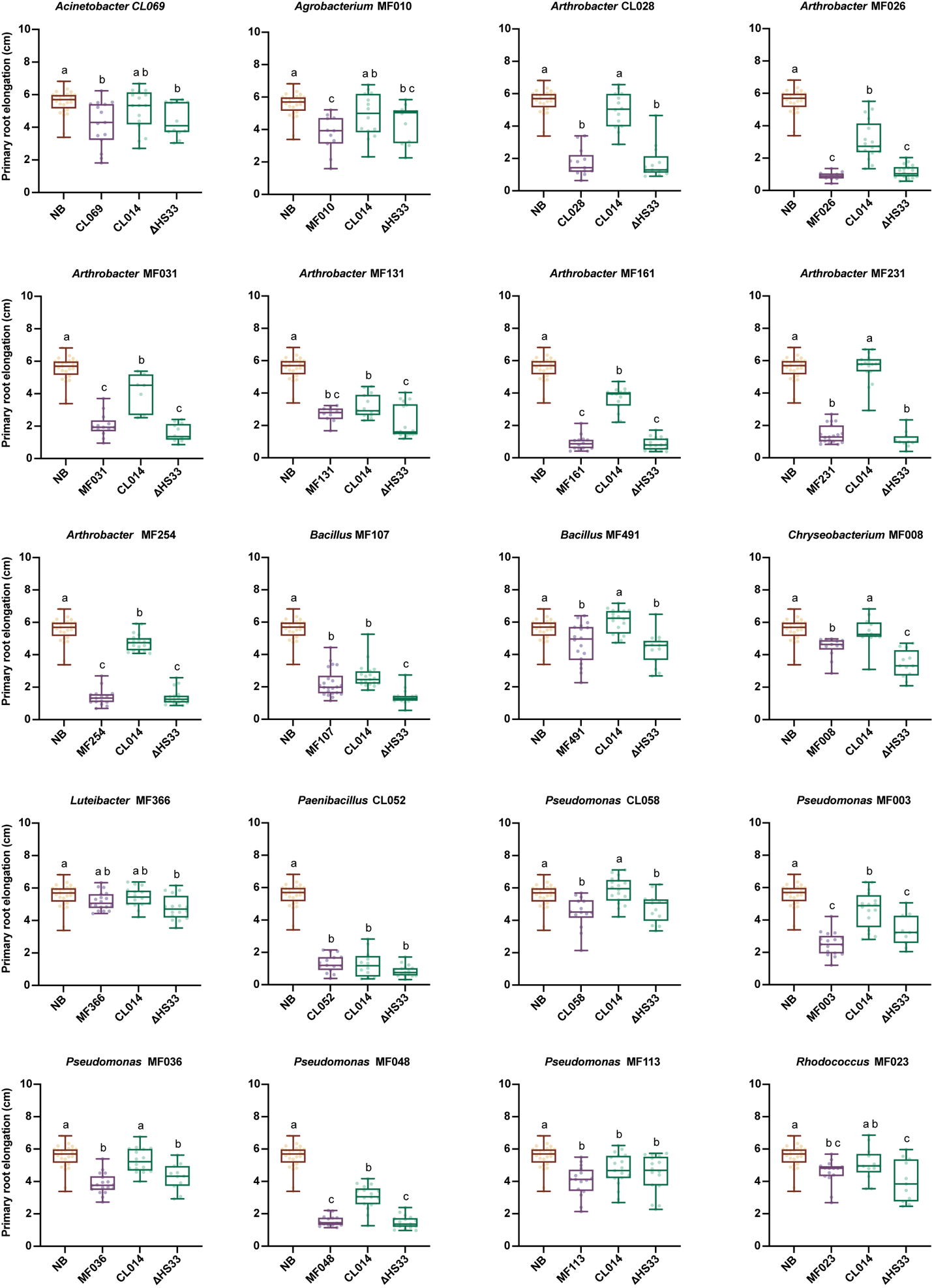
**

**Extended Data Fig. 7| Primary root length of *Arabidopsis* seedlings inoculated with previously identified RGI-inducing strains**^5^ **either alone (self) or co-inoculated with *V. paradoxus* CL014 wild type or the *iad*-deficient mutant ΔHS33, to assess *iad*-dependent reversal of root growth inhibition.**  Data were analyzed using one-way ANOVA with Tukey’s post hoc test; different letters indicate statistically distinct groups. “NB” indicates the no-bacteria control. Sample sizes (left to right, top to bottom): *n* = 28, 15, 16, 12, 28, 11, 14, 12, 28, 13, 14, 13, 28, 11, 17, 18, 28, 13, 7,12, 28, 10, 14, 18, 28, 14, 15, 17, 28, 19, 18, 11, 28, 16, 20, 18, 28, 22, 19, 16, 28, 18, 18, 15, 28, 13, 12, 12, 28, 18, 16, 16, 28, 15, 12, 17, 28, 14, 18, 13, 28, 17, 16, 11, 28, 18, 16, 11, 28, 14, 17, 17, 28, 16, 19, 16, 28, 15, 13, 11. Box plots display the median (center line), interquartile range (box), and whiskers extending to 1.5× the interquartile range.

**
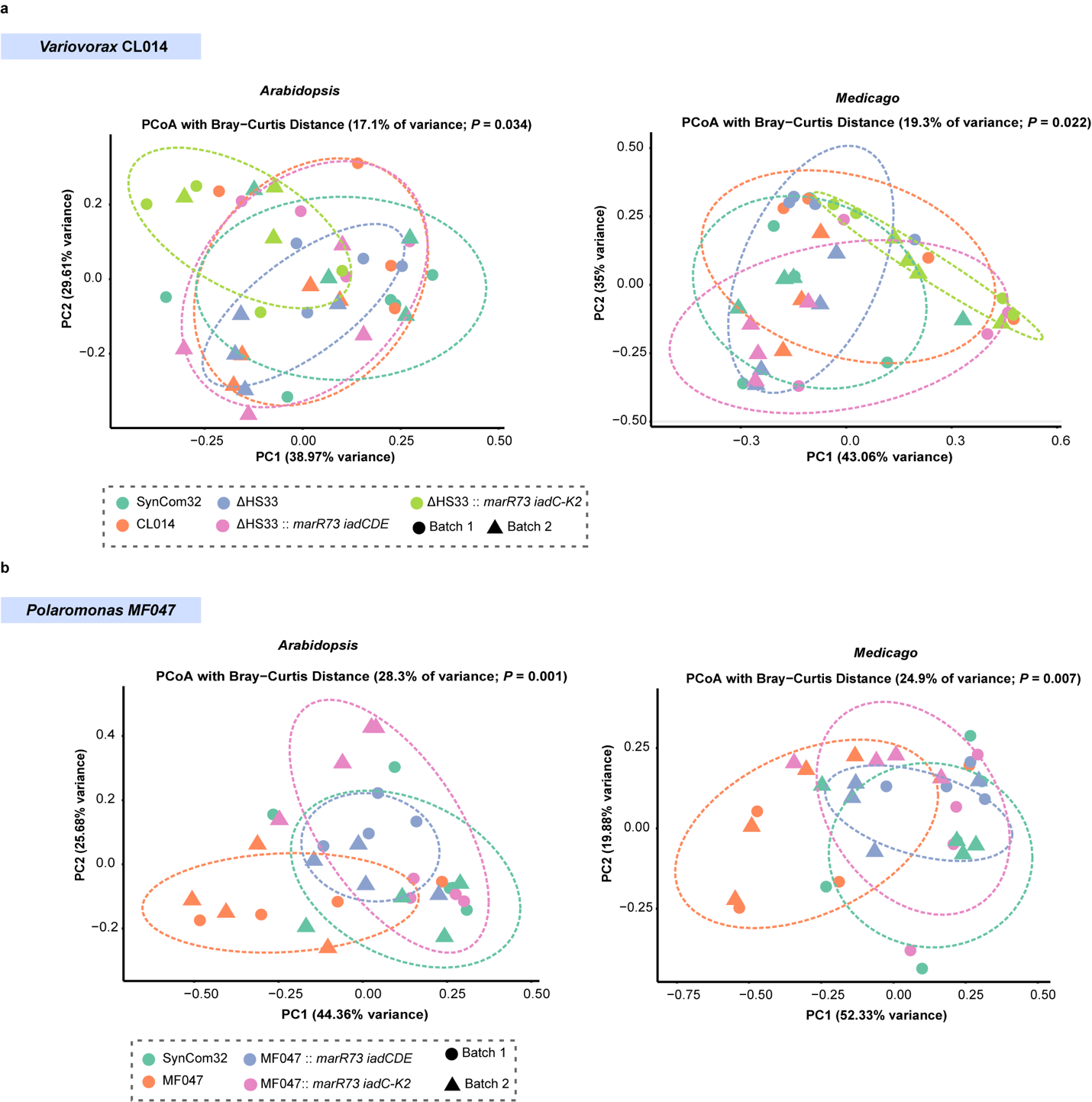
**E**xtended Data Fig. 8 | *V. paradoxus* CL014, *Polaromonas* MF047, and their engineered strains exhibit modest effects on root growth (17.1–28.3%, *P* = 0.05), consistent with their lower colonization levels.** Nonconstrained principal coordinate analysis (PCoA) of Bray–Curtis dissimilarity showing root microbiome composition in *Arabidopsis* and *Medicago* seedlings treated with SynCom32 alone or co-inoculated with engineered strains of *V. paradoxus* CL014 (**a**) and *Polaromonas* MF047 (**b**). Ellipses indicate 68% confidence intervals for each treatment group. Statistical significance was evaluated using PERMANOVA (Adonis2).


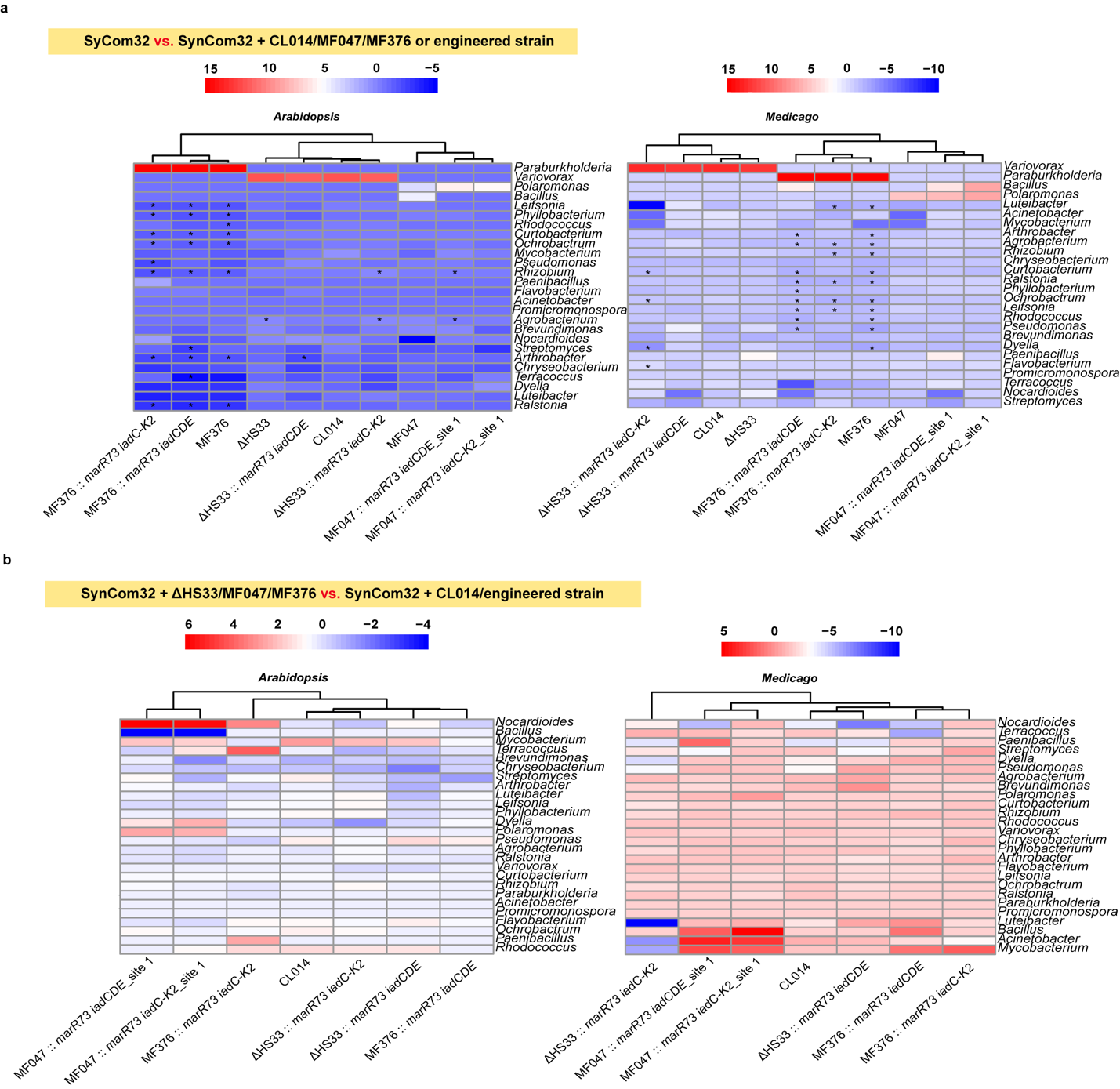
**Extended Data Fig. 9 | Log₂ fold-change analysis of genus-level shifts in *Arabidopsis* and *Medicago* root microbiomes.** Heatmaps show log₂ fold-change in bacterial genera abundance in root microbiomes treated with SynCom32 alone or co-inoculated with *V. paradoxus* CL014, *Polaromonas* MF047, *Paraburkholderia* MF376, or their engineered strains. **a**, Relative abundance changes compared to SynCom32 alone. **b**, Comparisons for each engineered strain group were made against different controls: SynCom32 + ΔHS33 for *V. paradoxus* CL014-related strains, SynCom32 + *Polaromonas* MF047 for *Polaromonas* MF047-related strains, and SynCom32 + *Paraburkholderia* MF376 for *Paraburkholderia* MF376-related strains. Pairwise comparisons were performed using Mann-Whitney U tests with FDR correction. Asterisks indicate significant differences (FDR-adjusted *P* < 0.05).


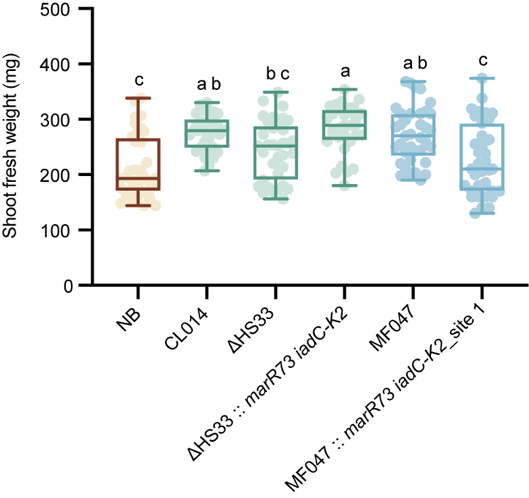


E**xtended Data Fig. 10 | Shoot biomass of 33-day-old *Arabidopsis* grown in natural soil inoculated with *V. paradoxus* CL014, *Polaromonas* MF047, and their engineered strains.** Shoot fresh weight of *Arabidopsis* plants grown for 33 days in natural soil treated with wild-type or engineered strains of *V. paradoxus* CL014 and *Polaromonas* MF047. Statistical significance was assessed by one-way ANOVA, with different letters indicating statistically distinct groups based on Tukey’s post hoc test. Sample sizes: *n* = 36, 34, 35, 32, 37, 35. Data were obtained from two independent experiments.
